# Orienting Attention to Short-Term Memory Representations via Sensory Modality and Semantic Category Retro-Cues

**DOI:** 10.1523/ENEURO.0018-20.2020

**Published:** 2020-11-24

**Authors:** Kristina C. Backer, Bradley R. Buchsbaum, Claude Alain

**Affiliations:** 1 Department of Cognitive and Information Sciences, University of California, Merced, CA 95343; 2 Rotman Research Institute at Baycrest Centre, Toronto, Ontario M6A 2E1, Canada; 3 Department of Psychology, University of Toronto, Toronto, Ontario M5S 3G3, Canada; 4 Institute of Medical Sciences, University of Toronto, Toronto, Ontario M5S 3G3, Canada; 5 School of Music, University of Toronto, Toronto, Ontario M5S 3G3, Canada

**Keywords:** attention, fMRI, memory, retro-cue, semantic

## Abstract

There is growing interest in characterizing the neural mechanisms underlying the interactions between attention and memory. Current theories posit that reflective attention to memory representations generally involves a fronto-parietal attentional control network. The present study aimed to test this idea by manipulating how a particular short-term memory (STM) representation is accessed, that is, based on its input sensory modality or semantic category, during functional magnetic resonance imaging (fMRI). Human participants performed a novel variant of the retro-cue paradigm, in which they were presented with both auditory and visual non-verbal stimuli followed by Modality, Semantic, or Uninformative retro-cues. Modality and, to a lesser extent, Semantic retro-cues facilitated response time relative to Uninformative retro-cues. The univariate and multivariate pattern analyses (MVPAs) of fMRI time-series revealed three key findings. First, the posterior parietal cortex (PPC), including portions of the intraparietal sulcus (IPS) and ventral angular gyrus (AG), had activation patterns that spatially overlapped for both modality-based and semantic-based reflective attention. Second, considering both the univariate and multivariate analyses, Semantic retro-cues were associated with a left-lateralized fronto-parietal network. Finally, the experimental design enabled us to examine how dividing attention cross-modally within STM modulates the brain regions involved in reflective attention. This analysis revealed that univariate activation within bilateral portions of the PPC increased when participants simultaneously attended both auditory and visual memory representations. Therefore, prefrontal and parietal regions are flexibly recruited during reflective attention, depending on the representational feature used to selectively access STM representations.

## Significance Statement

This functional magnetic resonance imaging study sought to examine similarities and differences in neural activity when concrete (sensory modality) and abstract (semantic category) information is used to guide attention to short-term memory representations of non-verbal stimuli. The posterior parietal cortex [PPC; especially portions of intraparietal sulcus and left ventral angular gyrus (AG)] had activation patterns that were specific to both modality-based and semantic-based reflective attention. Semantic-based reflective attention also recruited additional left-lateralized prefrontal regions and dorsolateral AG. Furthermore, dividing attention across sensory domains within memory was associated with stronger activation within the dorsomedial PPC. Thus, attentional orienting to memory flexibly recruits prefrontal and parietal regions as necessary, depending on the information used to selectively access memory representations.

## Introduction

It is well known that attention can not only be focused on external events (perceptual attention) but also be oriented toward internal representation(s) in short-term memory (STM; reflective attention; for review, see Chun and Johnson, 2011; Cowan, 2011; Gazzaley and Nobre, 2012; Backer and Alain, 2014; Souza and Oberauer, 2016). Like perceptual attention, reflective attention depends on a fronto-parietal attentional control network (Nobre et al., 2004; Nee and Jonides, 2009; Tamber-Rosenau et al., 2011) that plays an important role in selecting the attended representation, for example, within visual cortex (Kuo et al., 2014).

Neuroimaging studies on reflective attention often use a retro-cuing task in which a stimulus representation held in STM is cued retrospectively, that is, after the stimuli have been presented, during a retention interval. By manipulating which STM representation is retro-cued, prior studies have demonstrated that reflective attention modulates activation within the brain regions that hold the attended representation (Lepsien and Nobre, 2007; Lewis-Peacock et al., 2012; Han et al., 2013). For example, Lepsien and Nobre’s (2007) functional magnetic resonance imaging (fMRI) study revealed modulations of activation within the parahippocampal and fusiform gyri when participants reflectively attended a STM representation of a visual scene or face, respectively. Similarly, when participants reflectively attended spoken or written words, activity increased in the auditory or visual cortex, respectively (Buchsbaum et al., 2005; Johnson et al., 2005).

A complementary approach to further characterize the brain network enabling reflective attention is to manipulate how a representation is retro-cued, that is, manipulating the feature used to orient attention to a STM representation. For example, behavioral studies have shown performance benefits of retro-cuing a visual representation, based on its location or a non-spatial feature (e.g., color or shape; Pertzov et al., 2013; Li and Saiki, 2015; Heuer and Schubö, 2016; Ye et al., 2016). Using EEG, Backer et al. (2015) retro-cued listeners to one of four auditory STM representations, based on its spatial location or semantic category. The EEG results revealed common activity over fronto-central and posterior scalp sites, as well as ERP modulations over time depending on the cue used to orient attention to auditory memory. However, it remains unclear how the type of information used to selectively access a STM representation modulates the neural networks mediating reflective attention, because of EEG’s limited spatial resolution and the paucity of neuroimaging studies investigating this question.

The present fMRI study was designed to address this gap in knowledge. We created a novel variant of the retro-cue paradigm, which incorporated non-verbal auditory and visual stimulus arrays. We aimed to characterize brain activation when participants reflectively attended STM representations based on their sensory modality (auditory, visual) or semantic category (animal, music). The primary goal of this study was to determine the extent to which a common fronto-parietal attentional control network is engaged for both modality-based and semantic-based reflective attention.

Using fMRI, Cristescu et al. (2006) contrasted activity following pre-stimulus cues that guided attention to one word based on its spatial location or semantic category. Both semantic-based and spatial-based attentional orienting engaged bilateral parietal [intraparietal sulcus (IPS), superior parietal lobule (SPL)] and occipital cortex. Semantic pre-cues uniquely activated left inferior frontal gyrus (IFG), posterior temporal cortex, and angular gyrus (AG; Cristescu et al., 2006), regions that work with the dorsomedial prefrontal cortex (DMPFC) to mediate semantic processing more generally (Binder et al., 2009; Noonan et al., 2013). Thus, we hypothesized that semantic-based reflective attention to STM representations would more strongly engage PFC and parietal regions that underlie semantic processing, including the left DMPFC and AG, than modality-based reflective attention.

Previous studies have shown that the ventrolateral PFC (VLPFC) plays a role in selecting task-relevant memory representations, especially when competing task-irrelevant information is also present (Thompson-Schill et al., 1997; Johnson et al., 2005; Nee and Jonides, 2009). Thus, we expected to observe greater VLPFC activity following both Semantic and Modality retro-cues than Uninformative cue trials, which did not involve selectively attending a subset of STM representations. Furthermore, Ciaramelli et al. (2008) proposed that the IPS and SPL mediate top-down attention to episodic memory. If this mechanism generalizes to STM, then Semantic and Modality retro-cues should elicit stronger activation in the IPS and SPL than Uninformative retro-cues. Overall, our hypotheses point to a network of PFC and superior parietal regions that mediate reflective attention, regardless of the feature used to orient attention to a specific STM representation. Moreover, it is likely that additional brain regions are flexibly recruited depending on the feature used to access a particular STM representation.

## Materials and Methods

### Participants

Eighteen right-handed, young adult volunteers participated in this study. All participants self-reported normal or corrected-to-normal vision, normal color vision, and no history of neurologic or psychiatric disorders. MR-compatible glasses were provided to participants who wore their glasses to the session. Each participant completed an audiogram; all participants had normal pure-tone thresholds of ≤25-dB hearing level (HL) at each octave frequency from 250 to 8000 Hz in both ears. Datasets from two participants were excluded from the analyses because of excessive head motion in the scanner, leaving 16 datasets [eight males, 23.3 (mean) ± 3.2 (SD) years; range: 18–30 years]. Each participant provided informed written consent before commencing the study and received a small honorarium for their participation. The study protocol was approved by the Research Ethics Board at Baycrest Centre and was conducted in accordance with the Declaration of Helsinki.

### Stimuli and task

**Figure 1.**
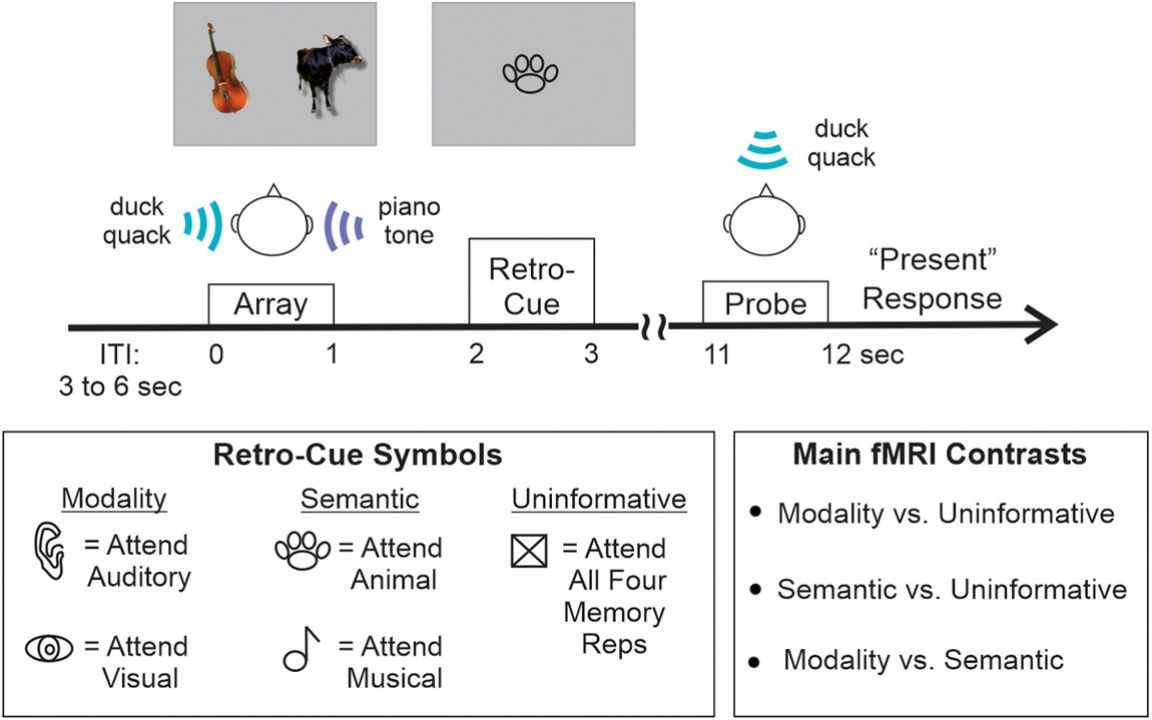
Experimental design. This figure depicts an example Animal retro-cue trial. Here, the participant would orient attention to the two animal representations (duck quack sound and cow picture) in memory and rehearse those two representations until the memory probe was presented. In this case, the probe was the duck quack sound; hence, the correct answer is “present.” Also illustrated are the symbols used for each retro-cue condition and a list of the main contrasts conducted on the fMRI data.

Figure 1 shows a schematic of a typical trial. Participants were presented with a bi-modal memory array, lasting 1005 ms and comprising two auditory and visual stimuli. After a brief delay (1000 ms), a retro-cue was visually presented at the center of the screen (1000 ms), followed by a long retention interval (8000 ms). The probe consisted of a single visual or auditory item (1005 ms). The retro-cue was either Informative or Uninformative. The Informative retro-cues indicated either the Modality (auditory or visual) or the Semantic category (animal or music) that was task-relevant. The retro-cue was a line drawing that indicated which items the participant should remember. There were five retro-cue conditions, including two Modality cues [ear (∼2.1° × 3.4° visual angle, width × height), eye (∼4.1° × 1.7°)], which instructed participants to focus attention on the two auditory or the two visual representations, respectively; two Semantic cues [paw (∼3.7° × 2.0°), musical note (∼2.8° × 2.7°)], which indicated that only the two animal or the two musical stimuli were task-relevant, respectively; and an Uninformative cue condition [box (∼3.0° × 2.4°)]. On trials with an Uninformative retro-cue, the probe could be a sound or a picture of any Semantic category. Informative retro-cues were always valid; for example, on Attend Auditory retro-cue trials, the probe was always a sound, and on Animal retro-cue trials, the probe was always an animal stimulus. This predictive relationship between the Informative retro-cues and probes was explained to all participants. As such, they were asked to only rehearse the cued representations on Informative retro-cue trials. The participants’ task was to judge whether the probe stimulus was present (50%) or absent (50%) within the memory array on each trial. Participants were instructed to respond to the probe as quickly and accurately as possible, via a button press. The intertrial interval (ITI) was jittered between 3 and 6 s (750-ms steps, rectangular distribution). Except when retro-cues or visual stimuli were on-screen, a central fixation cross was displayed on a gray background.

We chose 32 animal sounds and 32 musical sounds (all with 12207-Hz sampling rate, 1005-ms duration) from an in-house sound database. The sounds were normalized according to root mean square power, to approximately equate their loudness. Similarly, 32 pictures of animals and 32 pictures of musical instruments were selected and exported onto a gray background using Hemera PhotoObjects software. These images were normalized according to area (50,000 ± 500 pixels), using custom MATLAB (The MathWorks) code; the range in area was necessary to maintain the aspect ratio of the pictures.

Memory arrays (Fig. 1) were created by pairing two sounds with two pictures. Each picture and sound were used only once to create 32 memory arrays. The simultaneous presentation of two sounds with similar acoustic properties can mask one another. To mitigate masking, sound pairs were optimally selected with minimal spectrotemporal overlap. MATLAB code was used to first compute a spectrogram for each individual sound and then to conduct a two-dimensional cross-correlation for each possible sound pair. For each pair, the maximal cross-correlation value was recorded (since higher values indicate more similar spectrograms than lower values), and all possible pairings were sorted from least to most similar. We selected the 32 most optimal sound pairs with the following constraints: (1) each sound could be used just once; (2) there had to be 16 same-category pairs (eight animal-animal and eight musical-musical) and 16 mixed-category pairs (animal-musical); and (3) for the same-category pairs, two sounds within the same subcategory (e.g., two bird calls or two drumming sounds) could not be paired. We used these same three constraints for pairing picture stimuli together.

Next, these sound and picture pairs were combined to create the four-stimulus memory arrays, such that no auditory or visual stimuli within a memory array would correspond to the same animal or musical subcategory. For instance, a picture of a bird never occurred within the same memory array as a bird chirp. This was done to ensure that each memory array was always composed of four distinct objects. This pairing process yielded three memory array configurations: (1) two musical sounds and two animal pictures (eight arrays); (2) two animal sounds and two pictures of musical instruments (eight arrays); and (3) one animal sound, one musical sound, one animal picture, and one musical picture (16 arrays). The pictures in the memory arrays were presented on a gray background, one centered to the left and the other centered to the right of screen center; the two sounds were played dichotically through MRI-compatible headphones.

Each of the 32 memory arrays was presented five times in total, once per retro-cue condition. On trials with a Modality retro-cue, the two retro-cued items could be both musical stimuli (eight trials per retro-cue condition, i.e., ear and eye), both animal stimuli (eight trials per cue condition), or one item per category (16 trials per cue condition). Similarly, on Semantic retro-cue trials, the two retro-cued representations could be both visual (eight trials per cue condition, i.e., animal and musical), both auditory (right trials per cue condition), or one of each (16 trials per cue condition). The retro-cued items’ location (left/right), category, and modality were counterbalanced within participants.

The memory probe comprised one diotically-presented sound (perceived as coming from the center of the head) or one picture presented in the center of the screen. Each sound and picture comprising the 32 memory arrays was used as the memory probe once or twice. Stimuli within each sensory modality and category were used as the probe an equal number of times. On present-probe trials, the probe’s original location (left/right) was counterbalanced. On trials with an absent probe, the probe was a stimulus that was not heard or seen on that trial, and it could never involve a token change (e.g., a bird picture replaced by a different bird picture). Perfectly counterbalancing the probe’s modality, category, and location properties for the Uninformative cue trials required 64 trials; however, since there were only 32 Uninformative cue trials per participant, these 64 possible combinations were split into two sets of 32 probes, which were counterbalanced across participants.

Participants completed a practice run (20 trials) outside the scanner, with scanner noise played in the background to familiarize them with the scanning environment and task. Inside the scanner, they completed 8 runs, each comprising 20 trials and lasting 6 min 18 s. The trials were ordered pseudo-randomly, with the constraint that each run included four trials per retro-cue condition. Except for the first two participants, the trial order was further constrained, to maximally equate the probability of one retro-cue condition following another (or the same) condition.

During scanning, participants lay in the supine position. Visual stimuli were projected to a screen at the end of the scanner’s bore, and participants viewed the visual stimuli by looking into a mirror mounted onto the head coil. Auditory stimuli were delivered through MRI-compatible (MR Confon) headphones at ∼80-dB SPL; participants wore ear plugs as well, per standard scanning protocol. Several checks were done during the scanning session to ensure that participants could clearly hear and see the stimuli. Stimulus delivery was controlled with Presentation Software (Neurobehavioral Systems). In the scanner, participants responded by pressing a button on a response box, using their right index and middle fingers.

### Behavioral data analyses

The accuracy, d′, and response time (RT) data were analyzed using repeated measures ANOVAs [using R version 3.3.0 (Ihaka and Gentleman, 1996), with the ez Package: https://CRAN.R-project.org/package=ez], and paired-samples *t* tests were used to evaluate significant differences in accuracy, d′, and RT between the retro-cue conditions (for a list of all analyses, see Table 1). Custom MATLAB code was used to compute accuracy, d′, and RT from the raw behavioral data. Accuracy was computed as the percent correct, including both present and absent trials. For the d′ analysis, hit rates were calculated based on the present trials, and false alarm rates based on the absent trials. Because of ceiling performance in some participants, the hit and false alarms rates were adjusted by adding 0.5 to the number of hits and false alarms and adding 1 to the number of present and absent trials for each participant (Hautus, 1995), before computing d′. For the RT analysis, only correct trials with RTs of 3 s or less (relative to the memory probe onset) were included. Effect sizes are reported as η^2^ and generalized η^2^ (η^2^*_G_*) for the ANOVAs, and Cohen’s *d_z_* for the paired-samples *t* tests (Lakens, 2013). If the data violated the sphericity assumption (via Mauchly’s test), then Greenhouse–Geisser corrected *p* values were reported. *Post hoc* pairwise *t* tests, using Bonferroni correction, were performed if there was a significant main effect of retro-cue condition.

**Table 1:** Statistical table

Manuscript reference	Figure	Data type	Data structure	Type of test	Multiple comparisons correction	Program	Statistic	*p* value	Power or 95% confidence interval
a	2*A*	Accuracy	Normal distribution	One-way repeated-measures ANOVA		R	*F*_(2,30)_ = 1.25	0.29	η^2^ = 0.077, η^2^*_G_* = 0.028
b	2*B*	d'	Normal distribution	One-way repeated-measures ANOVA		R	*F*_(2,30)_ = 3.35	**0.049**	η^2^ = 0.18, η^2^*_G_* = 0.080
c			Normal distribution	*Post hoc* paired *t* test: Modality vs Uninformative	Bonferroni	R	*t*_(15)_ = 2.29	0.11	Mean difference = 0.38 95% CI: 0.027 to 0.73
d			Normal distribution	*Post hoc* paired *t* test: Semantic vs Uninformative	Bonferroni	R	*t*_(15)_ = 1.34	0.61	Mean difference = 0.22 95% CI: −0.13 to 0.56
e			Normal distribution	*Post hoc* paired *t* test: Modality vs Semantic	Bonferroni	R	*t*_(15)_ = 1.55	0.42	Mean difference = 0.16 95% CI: −0.060 to 0.38
f	2*C*	RT	Normal distribution	One-way repeated-measures ANOVA		R	*F*_(2,30)_ = 5.08	**0.013**	η^2^ = 0.25, η^2^*_G_* = 0.023
g			Normal distribution	*Post hoc* paired *t* test: Modality vs Uninformative	Bonferroni	R	*t*_(15)_ = −2.87	**0.035**	Mean difference = −62.88 95% CI: −109.57 to −16.20
h			Normal distribution	*Post hoc* paired *t* test: Semantic vs Uninformative	Bonferroni	R	*t*_(15)_ = −2.62	0.058	Mean difference = −45.10 95% CI: −81.78 to −8.41
I			Normal distribution	*Post hoc* paired *t* test: Modality vs Semantic	Bonferroni	R	*t*_(15)_ = −0.82	1.00	Mean difference = −17.79 95% CI: −63.76 to 28.18
j	3*A*	fMRI (BOLD)	No assumption	Paired *t* tests: Semantic > Uninformative	FWE-corrected non-parametric permutation test	FSL		
k		fMRI (BOLD)	No assumption	Paired *t* tests: Modality > Uninformative	FWE-corrected non-parametric permutation test	FSL		
l		fMRI (BOLD)	No assumption	Paired *t* tests: Uninformative > Semantic	FWE-corrected non-parametric permutation test	FSL		
m	3*B*	fMRI (BOLD)	No assumption	Paired *t* tests: Uninformative > Modality	FWE-corrected non-parametric permutation test	FSL		
n	4*A*	fMRI (BOLD)	No assumption	Paired *t* tests: Semantic > Modality	FWE-corrected non-parametric permutation test	FSL		
o	4*B*	fMRI (BOLD)	No assumption	Paired *t* tests: Modality > Semantic	FWE-corrected non-parametric permutation test	FSL		
p	5*A*	fMRI (BOLD)	No assumption	Paired *t* tests: Auditory > Visual	FWE-corrected non-parametric permutation test	FSL		
q	5*B*	fMRI (BOLD)	No assumption	Paired *t* tests: Visual > Auditory	FWE-corrected non-parametric permutation test	FSL		
r		fMRI (BOLD)	No assumption	Paired *t* tests: Animal > Musical	FWE-corrected non-parametric permutation test	FSL		
s		fMRI (BOLD)	No assumption	Paired *t* tests: Musical > Animal	FWE-corrected non-parametric permutation test	FSL		
t	6*A*	fMRI (MVPA AUC)	No assumption	One-sample *t* tests: Early Modality	FWE-corrected non-parametric permutation test	FSL		
u				One-sample *t* tests: Early Semantic		
v				One-sample *t* tests: Early Uninformative		
w	6*B*	fMRI (MVPA AUC)	No assumption	One-sample *t* tests: Late Modality	FWE-corrected non-parametric permutation test	FSL		
x				One-sample *t* tests: Late Semantic		
y				One-sample *t* tests: Late Uninformative		
z		Accuracy	Normal distribution	Paired *t* test: Intermodal vs Intramodal		R	*t*_(15)_ = −0.73	0.48	Cohen’s *d*_z_ = 0.18 Mean difference = −1.01 95% CI: −3.98 to 1.95
aa		d'	Normal distribution	Paired *t* test: Intermodal vs Intramodal		R	*t*_(15)_ = −0.49	0.63	Cohen’s *d*_z_ = 0.12 Mean difference = −0.081 95% CI: −0.43 to 0.27
ab		RT	Normal distribution	Paired *t* test: Intermodal vs Intramodal		R	*t*_(15)_ = 3.06	**0.0079**	Cohen’s *d*_z_ = 0.68 Mean difference = 27.50 95% CI: 8.37–46.64
ac	7	fMRI (BOLD)	No assumption	Paired *t* tests: Intermodal > Intramodal	FWE-corrected non-parametric permutation test	FSL		
ad		fMRI (BOLD)	No assumption	Paired *t* tests: Intramodal > Intermodal	FWE-corrected non-parametric permutation test	FSL		
ae		fMRI (BOLD)	No assumption	Paired *t* tests: Semantic-Both Auditory vs Attend Auditory cue	FWE-corrected non-parametric permutation test	FSL		
af		fMRI (BOLD)	No assumption	Paired *t* tests: Semantic-Both Visual vs Attend Visual cue	FWE-corrected non-parametric permutation test	FSL		
ag	8*A*, top	fMRI (BOLD): left dAG	Normal distribution	Paired *t* test: Modality vs Semantic	Bonferroni	R	*t*_(15)_ = −4.37	**0.0016**	Cohen’s *d*_z_ = 1.09 Mean difference = −0.90 95% CI: −1.34 to −0.46
ah			Normal distribution	Paired *t* test: Modality vs Uninformative	Bonferroni	R	*t*_(15)_ = −1.18	0.76	Cohen’s *d*_z_ = 0.30 Mean difference = −0.25 95% CI: −0.69 to 0.20
ai			Normal distribution	Paired *t* test: Semantic vs Uninformative	Bonferroni	R	*t*_(15)_ = 3.14	**0.020**	Cohen’s *d*_z_ = 0.78 Mean difference = 0.65 95% CI: 0.21 to 1.10
aj	8*B*, top	fMRI (BOLD): left vAG	Normal distribution	Paired *t* test: Modality vs Semantic	Bonferroni	R	*t*_(15)_ = 1.60	0.39	Cohen’s *d*_z_ = 0.39 Mean difference = 0.26 95% CI: −0.086 to 0.61
ak			Normal distribution	Paired *t* test: Modality vs Uninformative	Bonferroni	R	*t*_(15)_ = 3.13	**0.021**	Cohen’s *d*_z_ = 0.78 Mean difference = 0.84 95% CI: 0.27 to 1.40
al			Normal distribution	Paired *t* test: Semantic vs Uninformative	Bonferroni	R	*t*_(15)_ = 3.01	**0.027**	Cohen’s *d*_z_ = 0.75 Mean difference = 0.57 95% CI: 0.17 to 0.98
am	8*A*, lower	fMRI (BOLD): left dAG	Normal distribution	Paired *t* test: Intermodal vs Intramodal		R	*t*_(15)_ = 4.17	**0.00082**	Cohen’s *d*_z_ = 1.04 Mean difference = 0.60 95% CI: 0.30 to 0.91
an	8*B*, lower	fMRI (BOLD): left vAG	Normal distribution	Paired *t* test: Intermodal vs Intramodal		R	*t*_(15)_ = 2.18	**0.045**	Cohen’s *d*_z_ = 0.55 Mean difference = 0.36 95% CI: 0.0086 to 0.72

Significant *p*-values (< 0.05) are indicated in bold.

### fMRI data acquisition

Imaging data were collected using a Siemens Magnetom Trio 3.0-T scanner (Siemens Corporation) and a 12-channel head coil. First, high-resolution whole-brain magnetization prepared rapid gradient echo (MPRAGE) T1 images were obtained for anatomic localization [axial orientation, 160 slices, 1 mm thick; repetition time (TR): 2000 ms; echo time (TE): 2.63 ms; field of view (FOV): 192 × 256 mm]. Next, T2* functional images were collected, using a continuous single-shot, echoplanar imaging (EPI) acquisition sequence for blood oxygen level-dependent (BOLD) fMRI (33 axial slices, 3.5 mm thick with a 0.5-mm interslice gap; TR: 2000 ms; TE: 30 ms; flip angle: 70°; FOV: 225 × 225 mm; acquisition matrix: 96 × 96 voxels, resulting in an in-plane resolution of 2.34 × 2.34 mm). A total of 186 EPI volumes were collected during each run; the first five volumes were acquired during rest, allowing tissue magnetization stabilization.

### fMRI image preprocessing

Analysis of Functional NeuroImages (AFNI) software (Cox, 1996) was used to preprocess the brain images. First, the data were converted from DICOM to NIfTI-1 format. Next, motion and slice timing correction were done, realigning the images to the first run’s mean image volume, using the AFNI program *3dvolreg*. Each participant’s mean EPI image from the first run was co-registered to their MPRAGE using AFNI’s *3dAllineate* using the local Pearson cost function via the *align_epi_anat.py* script. The functional images were then smoothed with a 5 mm FWHM Gaussian smoothing kernel (*3dmerge*) for univariate GLM analyses. Finally, each participant’s MPRAGE image was nonlinearly transformed to a Montreal Neurologic Institute (MNI) template (bundled in FSL 5.0) with Advanced Normalization Tools software (ANTs; Avants et al., 2011). This transformation was later used to warp each participant’s univariate statistic maps to MNI space.

### fMRI: univariate analyses

A combination of R code, AFNI, and FSL (Smith et al., 2004) software was used to analyze the neuroimaging data. Using AFNI’s *3dDeconvolve* command, a whole-brain multiple-regression model was run on each participant’s smoothed EPI data (in each participant’s original EPI space). SPM’s canonical hemodynamic response function (HRF; Friston et al., 1994) was convolved with the data starting at the retro-cue onset and continuing for seven more seconds (i.e., during the task’s delay between the retro-cue and memory probe); this was done for each possible combination of the retro-cue condition, cued modality, and cued category, to create all of the contrasts of interest, described below. Additional regressors were added for the encoding (i.e., memory array) and recognition (i.e., memory probe) phases of each trial, but only the results from the retro-cue phase are reported here. Eight nuisance regressors per scanning run were entered into the model; three of these regressors were the first three principal components of matrix of motion parameter estimates (*x*, *y*, *z*, roll, pitch, yaw) output by *3dvolreg*, and five were derived from a principal component analysis restricted to the voxels with the highest 1% temporal variance (Behzadi et al., 2007). Since accuracy was very high on this task, all trials were included in the fMRI analyses. At the participants-level of the analysis, contrast volumes, containing the *t* statistics for each voxel, were extracted in line with our research questions, as detailed below. These single-participant univariate maps were then warped to MNI space, using the EPI to MNI transformation that was previously computed for each participant’s data.

Permutation tests were used for the group-level statistics. First, the single-participant *t* statistic contrast images were concatenated into a 4-dimensional image (with participants as the fourth dimension), using *fslmerge*. FSL’s permutation testing algorithm (*randomise*; Winkler et al., 2014) was used to create the null distribution data via random sign flips (10,000 permutations) and perform one-sample *t* tests on the 4D images. Since *randomise* only tests for significant effects among voxel values >0 (i.e., corresponding to the positive tail in each of our 4D contrast images), we also multiplied these 4D contrast images by −1 to test the voxel values corresponding to the negative tail of each contrast. Each contrast’s positive-tail and negative-tail 4-D volumes were inputted, one-by-one, into FSL’s *randomise* algorithm. The threshold-free cluster enhancement (TFCE; Smith and Nichols, 2009) option was used; also, 5-mm HWHM variance smoothing was performed, as recommended in FSL’s documentation for one-sample permutation tests on fewer than 20 participants. For each tail of each contrast, the *randomise* algorithm with the TFCE option resulted in three output volumes: (1) a raw *t* statistic volume; (2) a volume with uncorrected *p* values; and (3) a volume with family-wise error (FWE) corrected *p* values. Next, for each tail of each contrast, the *t* statistic volume was masked with the FWE-corrected *p* value volume (using *fslmaths* command), using an α threshold of 0.025, to correct for examining each contrast’s tail separately. These thresholded volumes were used to compile the tables with the fMRI results presented below. The FWE-corrected *p* value images were projected to surface maps for data visualization (i.e., the univariate fMRI results figures) using AFNI’s SUMA program (Saad and Reynolds, 2012).

Three primary contrasts were conducted: (1) Modality (collapsed across ear and eye cues) versus Uninformative retro-cues; (2) Semantic (collapsed across animal and musical cues) versus Uninformative retro-cues. The main purpose of these two contrasts, and their conjunction if applicable, was to determine the extent to which a common fronto-parietal attentional control network is engaged for both modality-based and semantic-based reflective attention. The third contrast, Modality (collapsed across ear and eye) versus Semantic (collapsed across animal and musical) retro-cues, was done to determine whether accessing a representation via different information domains reveals domain-specific activity, especially within the PFC and parietal cortex. (Table 1 contains a complete list of all fMRI contrasts.)

We also modeled the time course of the BOLD response across the whole brain and extracted these time courses within each significant cluster for each of the contrasts [i.e., functional regions of interest (ROIs)]. This was done in AFNI (*3dDeconvolve*) using a nine-parameter cubic spline function expansion, to model the impulse response from 0 to 18 s relative to the onset of the memory array, for each participant and retro-cue condition. Each participant’s estimated time course images were then converted from AFNI to NIfTI format and warped to MNI space, using the EPI to MNI transformation that was previously computed. Next, AFNI’s *3dmaskave* program outputted each participant’s average impulse response magnitude at each time step within each functional ROI, and for the appropriate retro-cue conditions for each contrast. The group-average time courses are shown in the fMRI results figures.

To foreshadow the univariate results, we observed robust activation differences in the AG for several contrasts. Prior studies have dissociated the functions of the ventral and dorsal AG during semantic processing (Seghier et al., 2010; Noonan et al., 2013). Seghier et al. (2010) proposed that the left dorsomedial AG mediates semantic search even when viewing non-semantic stimuli, and the left ventrolateral AG mediates conceptual identification of visual stimuli. Noonan et al. (2013) proposed that the left dorsal AG is part of a semantic control network and is sensitive to executive control demands, while the left ventral AG is engaged during semantic processing regardless of executive demands. Thus, we conducted an exploratory follow-up ROI analysis to more closely examine the observed whole-brain univariate activity in these ventral and dorsal AG subregions, during reflective attention.

Using AFNI, we created a sphere with an 8 mm radius centered on the left dorsomedial AG (−30, −66, 42) and left ventrolateral AG (−48, −68, 20); these MNI coordinates were from Seghier et al., 2010. These ROI spheres were resampled to the voxel resolution of our functional data, leading to 81 voxels per ROI. Next, we extracted the BOLD time courses for each participant and retro-cue condition (Modality, Semantic, Uninformative) and averaged across the BOLD values from 6 to 9 s (i.e., the early phase of the retention interval, accounting for hemodynamic lag). To test for differences between conditions, we conducted pairwise comparisons (i.e., paired *t* tests with Bonferroni correction) on these BOLD values, for each ROI separately.

### fMRI: multivariate pattern analysis (MVPA)

To complement the univariate analyses, we also performed a whole-brain searchlight analysis, a form of MVPA, on the fMRI images (Kriegeskorte et al., 2006). Whereas univariate analyses can reveal the level of brain activation associated with different retro-cue conditions, MVPA decodes brain activity to elucidate distributed patterns of activity that carry content-specific information. Here, we used MVPA to reveal patterns of activity that reliably discriminated between the retro-cue conditions.

Before beginning the searchlight analysis, the realigned unsmoothed fMRI images were extracted at a sequence of time points by linearly interpolating the time-series in 1-s increments, from −1 s to +14 s relative to memory array onset. The whole-brain searchlight analysis involved classification training and testing within a sphere of voxels (8-mm radius) that was centered on each voxel, one by one, through the entire brain volume. The searchlight analysis was repeated separately for each time point (−1 to +14 s). It used a single multiclass classifier to discriminate the current retro-cue condition from the other retro-cue conditions at time *t*. To identify pattern information discriminating the five retro-cue conditions [Attend Auditory (ear), Attend Visual (eye), Animal, Music, and Uninformative], we used multiclass shrinkage discriminant analysis (SDA) implemented in R (sda package: https://cran.r-project.org/web/packages/sda/index.html; Zuber and Strimmer, 2009; Ahdesmäki and Strimmer, 2010). To evaluate classifier performance, we used a hold-one-run out cross-validation, where each classifier was successively trained on *n*–1 runs while holding out the *n*th run for testing. Classification performance was computed as area under the curve (AUC) for each retro-cue condition using the vector of five (one per class) posterior probabilities of the SDA classifier generated for the successive held out test runs, relative to all other retro-cue conditions. During classifier performance evaluation, since the AUC was comparing each current retro-cue condition against all other retro-cue conditions (i.e., a binary decision) at time *t*, the chance AUC was 0.5. The classifier training and testing was conducted within the same participant’s data, and this whole training-testing procedure was repeated for all participants, leading to whole-brain AUC images for each participant, retro-cue condition, and time point. These AUC images were normalized to MNI space for further statistical analyses.

For the statistical analysis, we divided the retention interval into two parts: an early portion and a late portion, to more closely examine how the information conveyed in local patterns of activity might change throughout the retention interval. Patterns of activity during the early phase should carry information about the cue itself, as well as information needed for attentional orienting to the retro-cued representations. Patterns of activity during the late phase should reflect the maintenance of the retro-cued representations and possibly anticipation of the memory probe. Considering the hemodynamic lag, we averaged across the normalized AUC images corresponding to 6–9 s after the array (i.e., 4–7 s after retro-cue onset) for early, and across 10–13 s (i.e., 8–11 s after retro-cue onset) for late. We also averaged across the ear and eye cues to create Modality retro-cue images, and across the animal and music cues to create Semantic retro-cue images for the early and late phases. This resulted in AUC images corresponding to three main retro-cue conditions for statistical analyses (Modality, Semantic, and Uninformative), for both the early and late phases.

Before statistical analysis, 0.5 (chance) was subtracted from each voxel within the AUC images. Additionally, for each participant’s images, the average white matter AUC value was computed, based on FSL’s MNI 152 standard-space T1 white matter probability map, in voxels with a white matter probability of 0.9 and greater. If this average white matter AUC value was >0, it was subtracted from that participant’s AUC image for that retro-cue condition and time phase (early or late) image. We did this to correct for any possible positive biases in classification performance arising from head motion or other global sources of variation that might be correlated with the task design.

These AUC images were then inputted into FSL’s permutation testing algorithm (*randomise*), to identify the voxels for each time phase (early, late) and retro-cue condition (Modality, Semantic, Uninformative) that had a significantly positive AUC value (i.e., greater than chance) at the group level. These one-sample *t* test results were thresholded with an FWE-corrected *p* value of 0.05. Montages showing the searchlight results were created in MRIcron software (https://www.nitrc.org/projects/mricron). We also created two conjunction images in AFNI (one for early, other for late) to isolate voxels that uniquely discriminated just one retro-cue condition from all others (“condition-specific” voxels), as well as voxels whose pattern activity in the searchlight neighborhood could be reliably decoded for two or more retro-cue conditions (“conjunction” voxels). We then extracted the time courses for each participant and retro-cue condition within each cluster corresponding to condition-specific voxels or to conjunction voxels. Several of these time courses are displayed with the MVPA results.

## Results

### Informative versus Uninformative retro-cues: behavioral and univariate fMRI results

**Figure 2.**
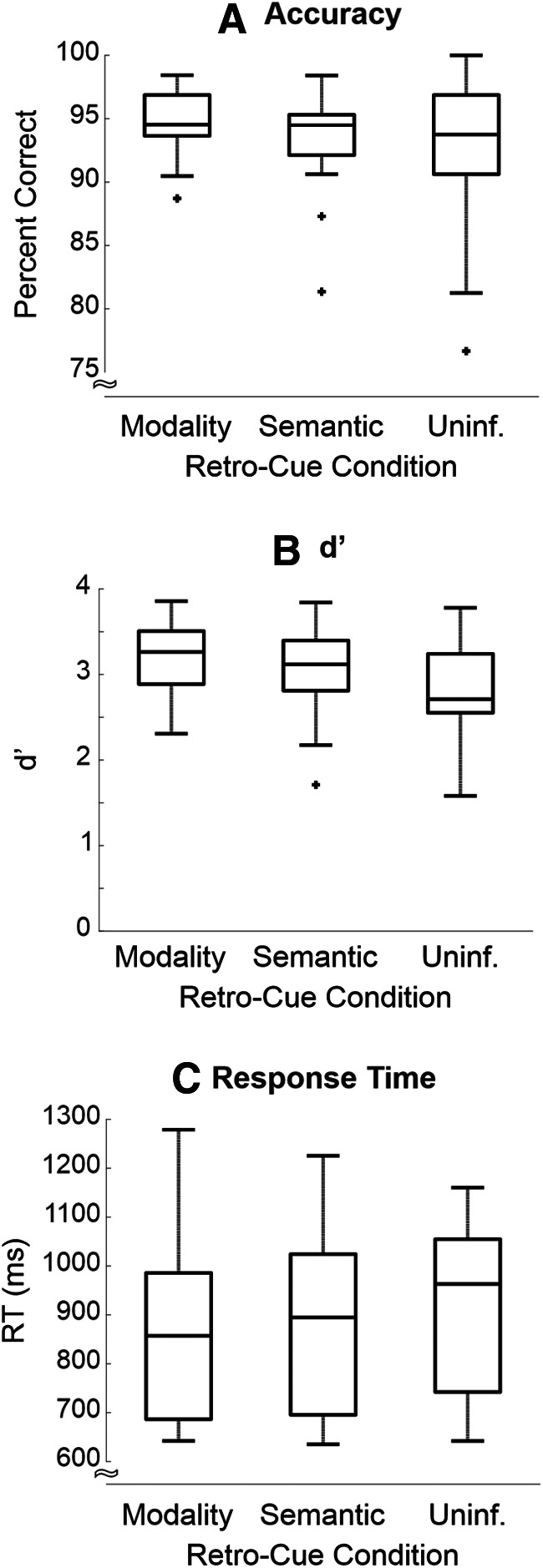
Behavioral results. Boxplots showing the group accuracy, d′, and RT. ***A***, Neither Modality nor Semantic retro-cues led to significant benefits in accuracy, relative to Uninformative cues. ***B***, However, there was a main effect of retro-cue condition on d′. ***C***, Furthermore, RT was faster for Modality cues and Semantic cues, relative to Uninformative cues.

First, we conducted three one-way repeated measures ANOVAs (one on accuracy data, one on d′ data, the third on RT data) to determine whether participants behaviorally benefited from Modality (collapsed across ear and eye retro-cue trials) and Semantic cues (collapsed across animal and musical retro-cue trials), relative to trials with Uninformative retro-cues. Figure 2 shows group mean accuracy (Fig. 2*A*), d′ (Fig. 2*B*), and RT (Fig. 2*C*). Overall, participants were very accurate, correctly identifying the probe as present or absent on 93% of trials. Participants’ accuracy was not affected by the retro-cue condition, *F*_(2,30)_ = 1.25, *p* = 0.29, η^2^ = 0.077, η^2^*_G_* = 0.028 (Table 1, a). A set of Shapiro–Wilk tests indicated that for the behavioral data, the accuracy values for the Semantic and Uninformative retro-cue conditions were not normally distributed (*p* = 0.011, *p* = 0.013, respectively). This non-normality was primarily driven by one participant’s data. The ANOVA for the accuracy data were re-run excluding that participant’s data, and the main effect of accuracy remained insignificant (*F*_(2,28)_ = 0.53, *p* = 0.60).

However, there was a significant main effect of retro-cue condition on d′, *F*_(2,30)_ = 3.35, *p* = 0.049, η^2^ = 0.18, η^2^*_G_* = 0.080 (Table 1, b). *Post hoc* pairwise comparisons (uncorrected) revealed that Modality retro-cues [mean (M) = 3.19; SE = 0.11] led to higher d′ than Uninformative retro-cues (M = 2.82; SE = 0.16; *p* = 0.037; evident in 10 of 16 participants), but there was no difference in d′ between Modality and Semantic retro-cues (M = 3.03; SE = 0.13; *p* = 0.14) or between Semantic and Uninformative retro-cues (*p* = 0.20). Upon applying Bonferroni correction, none of these pairwise differences in d′ were significant (Table 1, c–e). The most robust retro-cuing effect was observed for RT. Participants were quicker to respond when the retro-cue was Informative, *F*_(2,30)_ = 5.08, *p* = 0.013, η^2^ = 0.25, η^2^*_G_* = 0.023 (Table 1, f). *Post hoc* Bonferroni-corrected *t* tests (Table 1, g–i) indicated that participants were faster for both Modality (M = 859 ms; SE = 45 ms; *p* = 0.035; evident in 12 of 16 participants) and Semantic (M = 877 ms; SE = 46 ms, *p* = 0.058; observed in 14 of 16 participants) than for Uninformative retro-cues (M = 922 ms; SE = 42 ms). There was no difference in RT between Modality and Semantic retro-cue trials (*p* = 1.00). In summary, both types of Informative retro-cues facilitated RT, but not accuracy, relative to Uninformative retro-cues. The data also suggest a slight improvement in d′ following especially Modality retro-cues compared with Uninformative retro-cues.

**Figure 3.**
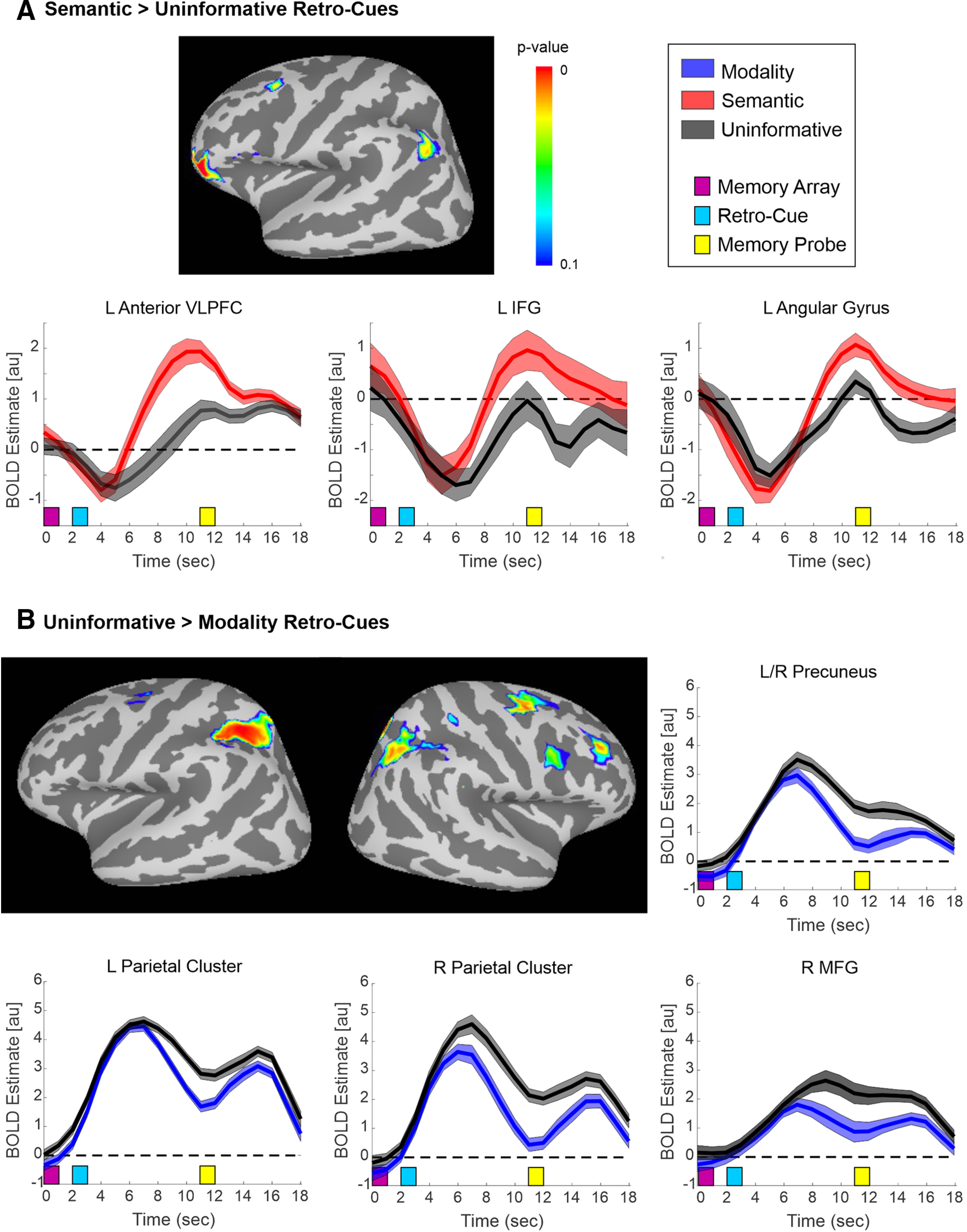
Univariate fMRI results for the contrasts: (***A***) Semantic > Uninformative retro-cues, (***B***) Uninformative > Modality retro-cues. Note that the opposite tails had no significant clusters. Surface maps are displayed, as well as BOLD time courses for selected significant clusters. For display purposes only, a liberal *p* value threshold of 0.10 (FWE-corrected) was used in this and subsequent univariate fMRI figures; however, for analysis, the data were thresholded at *p* < 0.025 (FWE-corrected). In this and subsequent univariate fMRI figures, clusters with a volume <50 mm^3^ are not shown on the surface maps, and the time courses have not been shifted to account for the hemodynamic lag. In this and subsequent figures (unless otherwise noted), the time courses show the group mean ± within-subjects SEM, which was calculated at each time point from the difference time course between the two displayed conditions. VLPFC, ventrolateral PFC; IFG, inferior frontal gyrus; MFG, middle frontal gyrus; au, arbitrary units.

The univariate fMRI results of the first two contrasts (Semantic vs Uninformative retro-cues; Modality vs Uninformative retro-cues; Table 1, j, k) are shown in Figure 3 and Tables 2, 3. These contrasts revealed activations that underlie selective reflective attention to two of the four items in STM. Significant clusters for Semantic > Uninformative cues comprised a left ventral fronto-parietal network, including the left anterior VLPFC, the left IFG, and the left AG. The time courses for these clusters showed similar initial deactivation for both Semantic and Uninformative retro-cues, followed by greater activation for the Semantic retro-cues. Notably, no significant clusters were observed for Modality > Uninformative cues.

**Table 2:** Univariate fMRI results: Semantic versus Uninformative retro-cues

Contrast	Brain area(s)	Cluster size	Max. T stat.	*x*, *y*, *z* (peak, mm)
Semantic > Uninformative	L anterior VLPFC	154	7.27	−46, 46, −6
	L AG	18	5.47	−49, −69, 40
	L IFG	10	4.76	−49, 25, −6

No significant clusters were observed for Uninformative > Semantic. In this table and in subsequent tables, cluster size is number of voxels, clusters with fewer than 10 voxels have been excluded, and coordinates are reported in MNI space.

**Table 3:** Univariate fMRI results: Uninformative versus Modality retro-cues

Contrast	Brain area(s)	Cluster size	Max. T stat.	*x*, *y*, *z* (peak, mm)
Uninformative > Modality	L/R precuneus, extending into L/R superior parietal gyrus	157	4.74	−1, −69, 52
	L IPS, L AG	157	5.61	−31, −51, 46
	R IPS, R AG	131	4.79	39, −57, 58
	R precentral sulcus, R superior frontal sulcus, extending into R MFG	63	5.17	33, −3, 52
	R MFG	39	5.47	42, 34, 34
	L superior parietal gyrus	15	4.20	−22, −69, 61

No significant clusters were observed for Modality > Uninformative.

By examining the opposite tail of these contrasts (Uninformative > Semantic retro-cues; Uninformative > Modality retro-cues; Table 1, l, m), we could determine which areas were more activated because of higher STM load (four vs two items). Surprisingly, no significant clusters were observed for Uninformative > Semantic retro-cues. However, Uninformative retro-cues led to greater activity in bilateral parietal cortex including bilateral precuneus, IPS, AG, and superior parietal gyrus than Modality retro-cues. Uninformative retro-cues also resulted in greater activity in right frontal cortex including middle frontal gyrus (MFG), precentral sulcus, and superior frontal sulcus. Please note that the univariate result images corresponding to these and subsequent significant univariate contrasts can be viewed on NeuroVault (Gorgolewski et al., 2015) at https://identifiers.org/neurovault.collection:9069.

### Semantic versus Modality retro-cues: univariate fMRI results

Next, we analyzed the fMRI data to determine whether the information domain used to reflectively attend to STM representations (i.e., input Modality or Semantic category) modulates the neural networks involved in orienting and sustaining attention to STM (Table 1, n, o).

**Figure 4.**
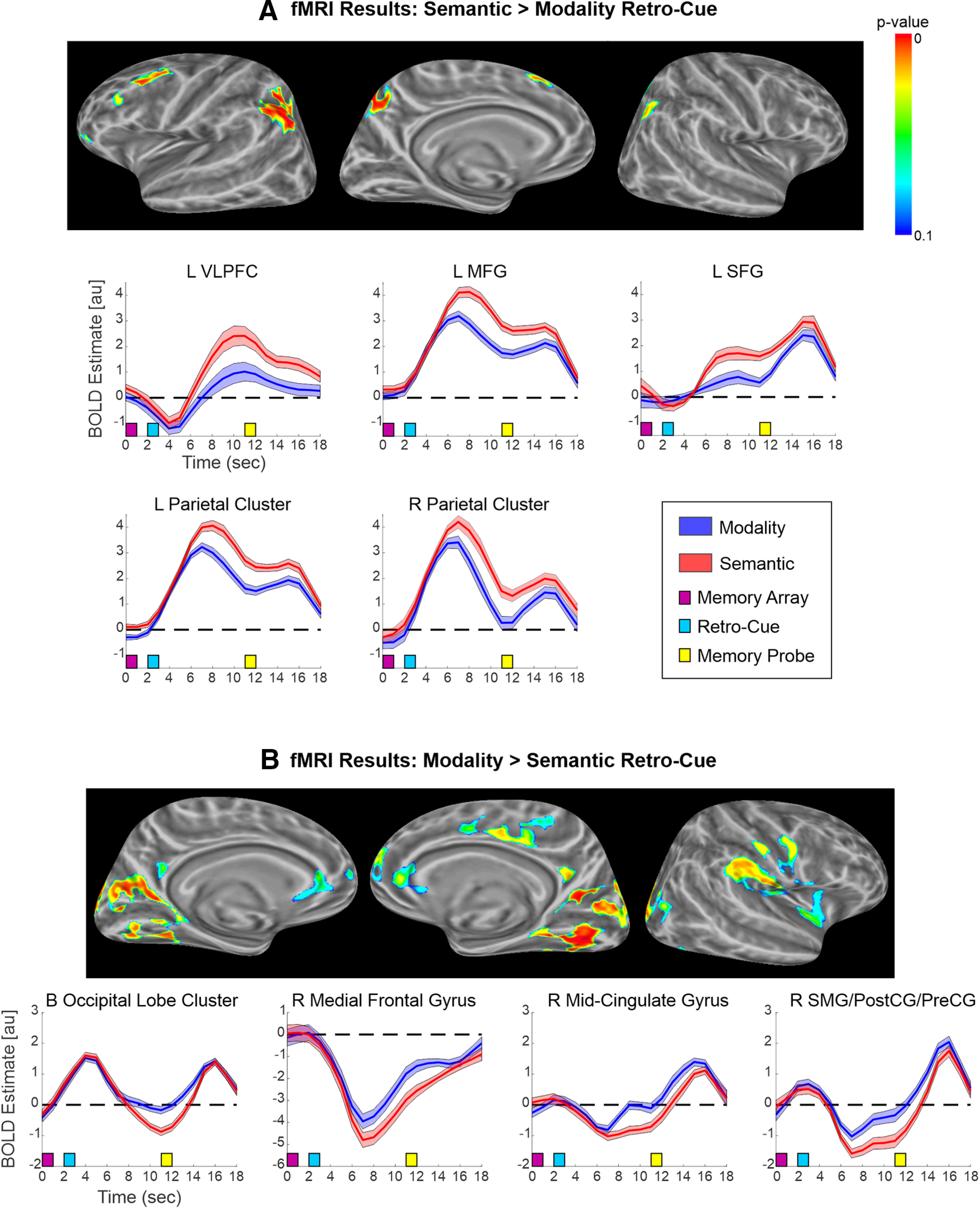
Univariate fMRI results for Semantic versus Modality retro-cues. Surface maps indicating clusters with stronger activity for (***A***) Semantic > Modality retro-cues and (***B***) Modality > Semantic retro-cues. For both tails of the contrast, BOLD time courses for the significant clusters are also displayed. Semantic retro-cues led to stronger activation in a left-lateralized fronto-parietal network, and stronger deactivation in a right-lateralized network. VLPFC, ventrolateral PFC; MFG, middle frontal gyrus; SFG, superior frontal gyrus; SMG, supramarginal gyrus; PostCG, postcentral gyrus; PreCG, precentral gyrus.

As displayed in Figure 4*A* and Table 4, when participants reflectively attended to Semantic category versus sensory Modality, significantly greater activation was observed in a left-lateralized frontal-parietal network, including left anterior VLPFC, MFG, superior frontal gyrus (SFG) within the DMPFC, and parietal cortex (IPS, AG, superior parietal cortex, and precuneus). There was additionally a cluster in the right parietal cortex (IPS and AG), although it covered a smaller area and was weaker than that in the left parietal cortex. Examining the BOLD time courses in each significant cluster revealed three different patterns of time courses, as shown in Figure 4*A*. First, the cluster in the left VLPFC (which overlapped with the VLPFC cluster observed in the Semantic vs Uninformative contrast; Fig. 3*A*) was initially deactivated after the memory array for both Semantic and Modality retro-cue trials, but following the retro-cue, activation increased to a greater extent for the Semantic retro-cues, approaching baseline after the probe. Second, the left MFG and bilateral parietal cortex, as well as in the right cerebellum (data not shown in Fig. 4) showed similar time courses. In these clusters, activation increased sharply after the retro-cue, followed by a decrease, and then a smaller increase after probe presentation; however, the initial increase was stronger following Semantic retro-cues, and sustained BOLD responses, from the retro-cue to probe presentation, were greater for the Semantic than Modality retro-cues. Finally, the BOLD response in the left SFG increased slightly after the retro-cue but to a greater extent for the Semantic than Modality retro-cues, followed by a sharp increase in amplitude for both conditions following the probe.

**Table 4:** Univariate fMRI results: Semantic versus Modality retro-cues

Contrast	Brain area(s)	Cluster size	Max. T stat.	*x*, *y*, *z* (peak, mm)
Semantic > Modality	L precuneus, L superior parietal gyrus, L IPS, L inferior parietal gyrus, L AG	626	6.63	−37, −60, 58
	L MFG	199	5.85	−52, 22, 31
	L/M SFG	44	6.12	−1, 37, 43
	R cerebellum	41	6.46	9, −81, −27
	L VLPFC	38	5.04	−43, 55, 1
	R IPS, R AG	31	4.24	33, −66, 49
	R cerebellum	18	6.05	36, −72, −54
Modality > Semantic	L/R superior and middle occipital gyri, L/R cuneus, L/R calcarine sulcus, L/R parieto-occipital fissure, L/R lingual gyrus, L/R fusiform gyrus, L/R collateral sulcus, R subparietal sulcus	1412	5.76	15, −72, −3
	R supramarginal gyrus, R postcentral gyrus, R central sulcus, R precentral gyrus	154	5.04	57, −18, 19
	R cingulate sulcus/gyrus	21	5.22	12, −9, 43
	R medial frontal gyrus	14	4.40	3, 58, 7

For the opposite tail of the contrast (i.e., Modality > Semantic retro-cues; Fig. 4*B*), significant activation differences were found in the occipital lobe: extending from the right lingual and fusiform gyri through bilateral middle/superior occipital gyri and into the left lingual and fusiform gyri. Significant clusters were also observed in the right lateral cortex (supramarginal gyrus, extending through postcentral gyrus and central sulcus to precentral gyrus), the right cingulate sulcus/gyrus, and the right medial frontal gyrus. The time courses for each of these clusters indicated that orienting attention to semantic objects led to stronger deactivation in these regions following retro-cue presentation, than reflective attention to sensory modality.

**Figure 5.**
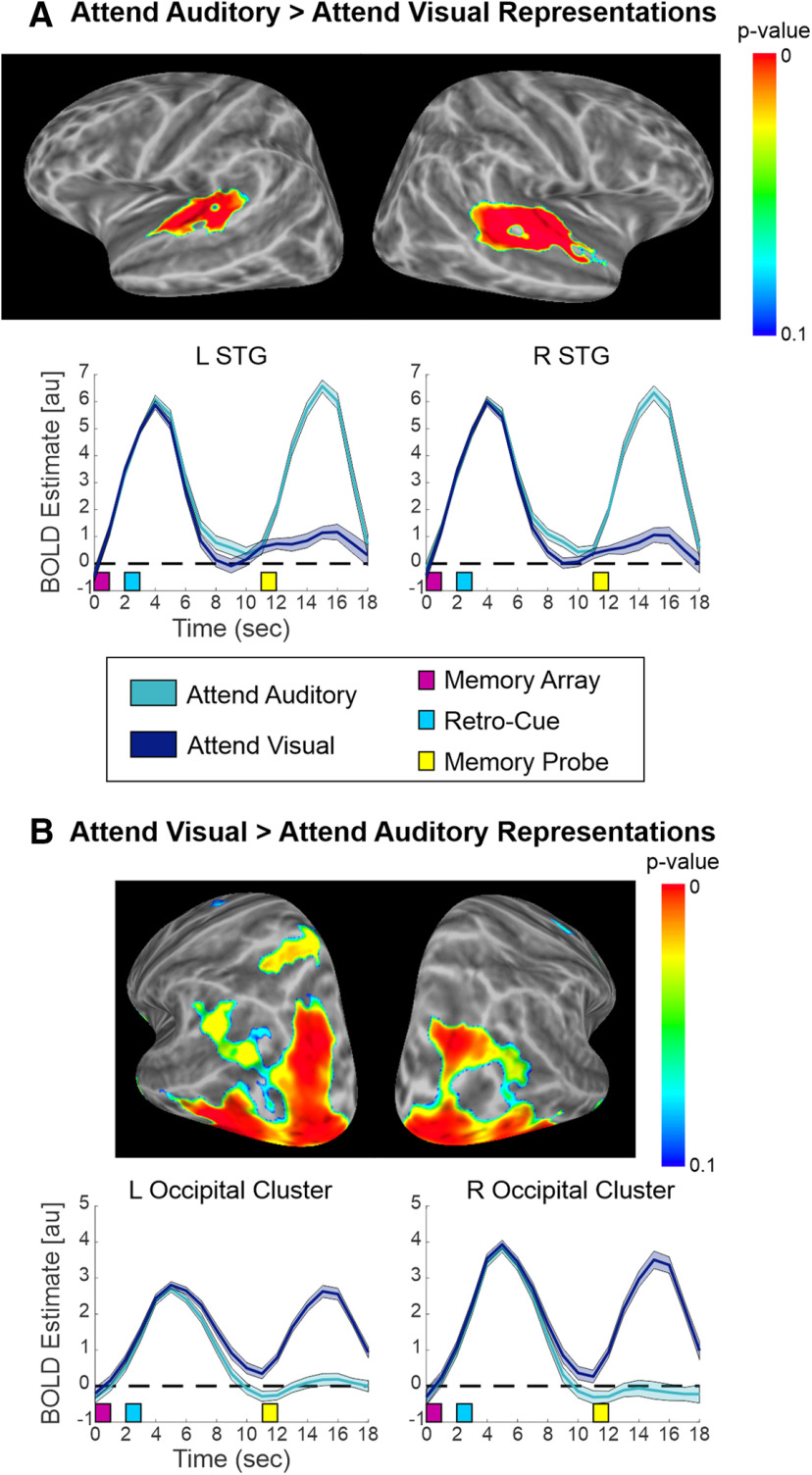
Attention to auditory versus visual STM. Surface maps indicating univariate clusters with stronger activity for (***A***) attention to auditory > visual STM and (***B***) attention to visual > auditory STM, highlighting the heightened response in the retro-cued modality’s sensory cortex. STG, superior temporal gyrus.

We also examined the fMRI data to determine whether there were differences in the BOLD response between retro-cues directing attention to auditory versus visual STM (Table 1, p, q), and between animal and musical retro-cues (Table 1, r, s). By contrasting brain activity following Attend Auditory versus Attend Visual retro-cues, we aimed to replicate and extend the findings of Buchsbaum et al. (2005) and Johnson et al. (2005) to non-verbal stimuli. In brief, we observed significant differences between auditory and visual retro-cue trials, such that there was greater activity in the sensory cortex of the retro-cued modality, as illustrated in Figure 5. Since this finding replicates Buchsbaum et al. (2005) and Johnson et al. (2005), we chose not to focus on it herein. Attention to visual representations was also associated with stronger activation in the bilateral medial temporal lobe (including hippocampus), medial PFC, left SFG, left ventral AG, and left IPS/superior parietal cortex than attention to auditory representations. There were no significant differences in the BOLD response between animal and musical retro-cues.

### MVPA results

**Figure 6.**
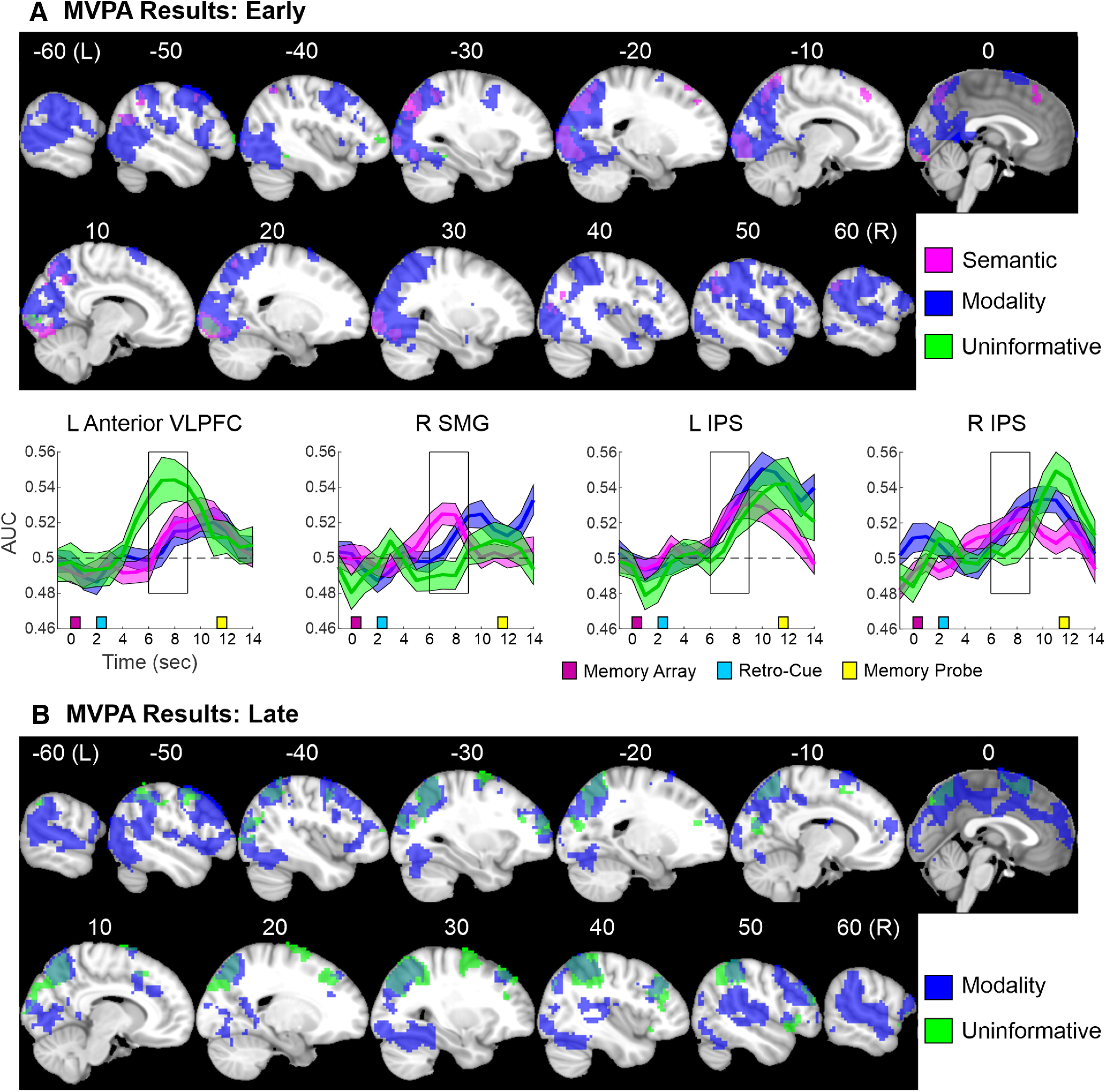
MVPA results. Montages display the MVPA results for the three main retro-cue conditions (Modality, Semantic, Uninformative) for the (***A***) early and (***B***) late phases of the retention interval. Time courses are shown for a few clusters. The left anterior VLPFC cluster had activation patterns that reliably distinguished the Uninformative cue condition from the other conditions, while the right SMG time course was derived from voxels whose pattern activity discriminated only the Semantic condition from the other conditions. The left and right IPS time courses were derived from voxels whose pattern activity encoded information related to both the Semantic and Modality conditions, but not the Uninformative condition. The box on the time course figures indicates the time points (6–9 s) included in the early time window. The within-subjects SEM displayed in the ribbon plots was computed on the mean-centered time courses for each subject. For the late phase, the MVPA did not successfully decode activation patterns related to the Semantic cue condition.

The MVPA searchlight analysis focused on decoding activity patterns during the retention interval that discriminated each retro-cue condition from the other retro-cue conditions. As previously described, each participant’s searchlight classification performance (AUC) images were collapsed across the Modality retro-cues (attend auditory and attend visual) and across the Semantic retro-cues (animal and music) before the permutation tests (Table 1, t–y). Figure 6*A* displays the MVPA results of the three retro-cue conditions (Modality, Semantic, Uninformative) during the early portion of the retention interval. Notably, the activity patterns related to Modality retro-cues could be reliably discriminated from the other conditions in a widespread region of neocortex. A conjunction analysis with the three retro-cue conditions revealed two clusters within the occipital lobe in which activity patterns during the early phase reliably dissociated each retro-cue condition from the others, likely reflecting the decoding of the visual cortex’s representation of the retro-cue symbols. Unlike the univariate results, the multivariate searchlight analysis revealed areas with activity patterns that reliably discriminated both the Semantic and Modality, but not Uninformative, retro-cues from the other retro-cue conditions. These areas included bilateral occipital regions and posterior parietal regions including the left and right IPS, AG, and precuneus/SPL, as well as a very small cluster in the left posterior MFG/precentral sulcus. This conjunction analysis further revealed brain regions with activity patterns that conveyed information about the Semantic retro-cues only, including the medial SFG and posterior parietal regions (left and right precuneus/SPL and AG, and right supramarginal gyrus). Furthermore, activity patterns in the left anterior VLPFC reliably discriminated only the Uninformative cue condition from the other cue conditions. This cluster partially overlapped with, but extended slightly dorso-medially relative to, the left anterior VLPFC clusters that were more active for Semantic than Uninformative and Modality retro-cues in the univariate analyses.

While activity patterns related to Semantic retro-cues could be reliably discriminated from the other conditions during the early phase, no brain areas had activity patterns that were specific to Semantic retro-cues during the late phase. However, during the late phase, activity patterns in prefrontal and parietal regions reliably conveyed information related to Modality and Uninformative cues (Fig. 6*B*). Moreover, activity patterns related to only Modality retro-cues could be reliably discriminated from the other conditions in bilateral STG and ventral visual regions, during the late phase.

### Intramodal versus Intermodal cuing effects: behavioral and univariate fMRI results

The previous univariate contrasts revealed increased activation within PFC and bilateral parietal cortex following both Semantic and Uninformative retro-cues relative to Modality retro-cues. Unlike the Modality retro-cues, which always guided attention to two STM representations of the same input modality, the Uninformative retro-cues required attending to two auditory and two visual representations. Furthermore, the Semantic retro-cues guided attention to two representations from the same input modality (both auditory or both visual) on 50% of the Semantic cue trials, and to two representations from different modalities (one auditory and one visual) on the other 50% of the trials. Thus, it is possible that reflective attention to different modalities on Uninformative and Semantic cue trials led to increased univariate activation in PFC and bilateral parietal cortex, relative to the Modality cue trials.

To directly test this idea, we divided the Semantic retro-cue trials according to whether the cue guided attention to two representations from the same sensory modality (Intramodal trials) or to two representations from different modalities (Intermodal trials, exemplified in Fig. 1). Crucially, both conditions (Intramodal and Intermodal) involved selecting and maintaining two representations in STM, regardless of the number of cued modalities. Behavioral and fMRI data were analyzed, contrasting the Intramodal and Intermodal trials.

Regarding behavioral performance, separate *t* tests were conducted on the accuracy, d′, and RT data. A paired *t* test showed no effect on accuracy (*t*_(15)_ = −0.73, *p* = 0.48, Cohen’s *d*_z_ = 0.18; Table 1, z) or d′ (*t*_(15)_ = −0.49, *p* = 0.63, Cohen’s *d*_z_ = 0.12; Table 1, aa). However, participants responded significantly faster for the Intramodal condition (i.e., when the cued representations were from the same sensory modality; M = 863 ms; SE = 44 ms) than the Intermodal condition (i.e., when the cued representations were from different modalities; M = 890 ms; SE = 48 ms; *t*_(15)_ = 3.06, *p* = 0.008, Cohen’s *d*_z_ = 0.68; Table 1, ab); 12 of 16 participants showed this pattern of results.

**Figure 7.**
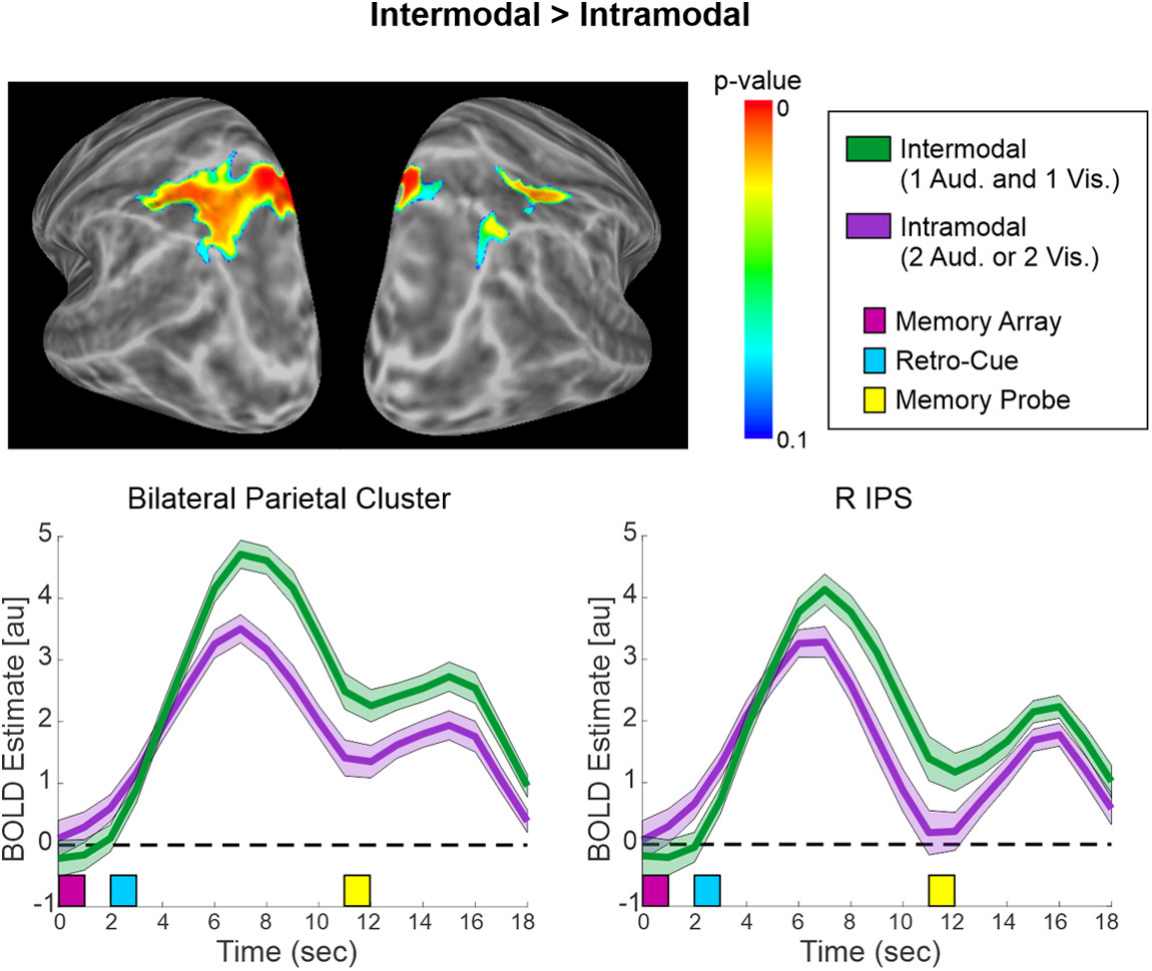
Intermodal versus Intramodal semantic retro-cue trials. Univariate fMRI results for the contrast between Intermodal (cued to one auditory and one visual STM representation) > Intramodal (cued to two auditory or two visual STM representations) trials. The surface maps illustrate enhanced activity in left-lateralized parietal cortex, and the BOLD time courses within the significant clusters are displayed. Note that there were no significant clusters for the other tail of this contrast (Intramodal > Intermodal).

Regarding the fMRI data, a whole-brain univariate contrast was conducted between the Intramodal and Intermodal Semantic retro-cue trials (Table 1, ac, ad). The analysis methods were identical to those used for the previous fMRI contrasts. The Intermodal trials (i.e., cued to one auditory and one visual representation) showed increased activity in the parietal lobe (i.e., bilateral precuneus, extending into left IPS, left AG, and bilateral superior parietal gyrus; and right IPS) compared with the Intramodal trials (i.e., cued to either two auditory or two visual representations; Fig. 7**;** Table 5). Upon examination of the BOLD time courses, both parietal clusters exhibited a similar pattern, with the Intermodal condition showing a more positive BOLD response than the Intramodal condition. The time courses were similar in the Intramodal condition, regardless of whether two visual or two auditory representations were attended (data not shown). No activation differences were found within the PFC. There were no significant clusters for the other tail of this contrast, Intramodal > Intermodal.

**Table 5:** Univariate fMRI results: Intermodal semantic retro-cue trials versus Intramodal semantic retro-cue trials

Contrast	Brain area(s)	Cluster size	Max. T stat.	*x*, *y*, *z* (peak, mm)
Intermodal > Intramodal	L/R precuneus, L/R superior parietal gyrus, L IPS, L AG	635	5.46	−1, −69, 49
	R IPS	94	4.68	42, −54, 58

There were no significant clusters for the other tail of the contrast: intramodal > intermodal.

We conducted a follow-up univariate fMRI analysis to contrast the Attend Auditory and Attend Visual Modality retro-cue trials with the Semantic-Both Auditory and Semantic-Both Visual retro-cue trials, respectively (Table 1, ae and af). However, only the contrast Semantic-Both Visual > Attend Visual yielded significant results, comprising several small clusters (23 voxels and fewer) in the left inferior frontal sulcus, left posterior parietal cortex (PPC; dorsal AG, IPS, SPL, precuneus), and right cerebellum.

### ROI analysis: left dorsal and ventral AG

**Figure 8.**
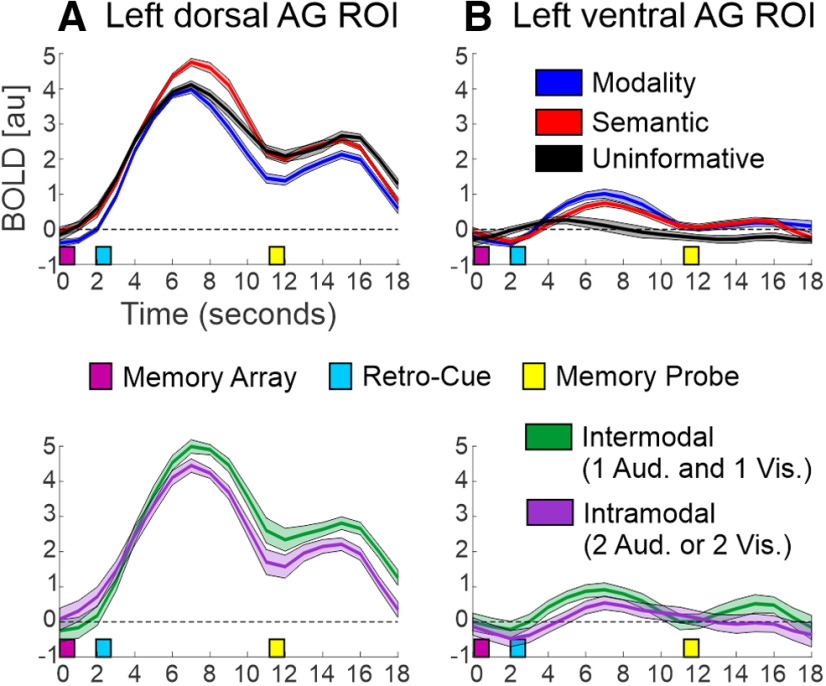
Univariate BOLD time courses in (***A***) left dorsal and (***B***) left ventral AG ROIs. The top row shows the time courses for the three main retro-cue conditions (Modality, Semantic, Uninformative), with the ribbons displaying the group mean time course ± the mean-centered within-subjects SEM. The bottom row shows the time courses separately for the Intermodal and Intramodal semantic retro-cue trials, with the ribbons depicting the group mean time course ± SEM of the within-subjects difference between the Intermodal and Intramodal conditions.

An exploratory ROI analysis was conducted to more closely examine univariate activation in the left dorsal and ventral AG, during the early phase of the retention interval following the retro-cue. Figure 8 depicts the full BOLD time courses for each condition and ROI. In the left dorsal AG ROI, the Semantic retro-cues (M = 4.45; SE = 0.43) showed a stronger BOLD response than both the Modality (M = 3.55; SE = 0.39; *t*_(15)_ = −4.37, *p* = 0.0016, Cohen’s *d*_z_ = 1.09; Table 1, ag) and Uninformative (M = 3.80; SE = 0.37; *t*_(15)_ = 3.14, *p* = 0.020, Cohen’s *d*_z_ = 0.78; Table 1, ai) retro-cues. There was no difference between the Modality and Uninformative conditions (*t*_(15)_ = 1.18, *p* = 0.76, Cohen’s *d*_z_ = 0.30; Table 1, ah). In the left ventral AG ROI, both the Modality (M = 0.90; SE = 0.49; *t*_(15)_ =3.13, *p* = 0.021, Cohen’s *d*_z_ = 0.78; Table 1, ak) and Semantic (M = 0.63; SE = 0.48; *t*_(15)_ = 3.01, *p* = 0.027, Cohen’s *d*_z_ = 0.75; Table 1, al) retro-cues showed a stronger BOLD response than the Uninformative retro-cues (M = 0.060; SE = 0.44). There was no difference between the Semantic and Modality cues (*t*_(15)_ = 1.60, *p* = 0.39, Cohen’s *d*_z_ = 0.39; Table 1, aj).

To determine whether the Semantic retro-cue effects were driven by the Intermodal trials, we repeated this analysis on the Intermodal and Intramodal trials. This revealed that Intermodal trials (dorsal AG: mean = 4.72, SE = 0.47; ventral AG: mean = 0.80; SE = 0.51) had a stronger BOLD response in the dorsal AG (*t*_(15)_ = 4.17, *p* = 0.00082 (uncorrected), Cohen’s *d*_z_ = 1.04; Table 1, am), and to a lesser extent in the ventral AG [*t*_(15)_ = 2.18, *p* = 0.045 (uncorrected), Cohen’s *d*_z_ = 0.55; Table 1, an] compared with the Intramodal trials (dorsal AG: mean = 4.12, SE = 0.41; ventral AG: mean = 0.43; SE = 0.48).

## Discussion

This study sought to investigate the extent to which modality-based and semantic-based reflective attention rely on a common attentional control network. To do so, we designed a novel variant of the retro-cue paradigm to manipulate how STM representations are selectively accessed, that is, based on their sensory modality (auditory, visual) or semantic category (animal, music). Overall, the fMRI results revealed modulations in prefrontal and parietal regions, consistent with prior research on interactions between top-down attention and memory (for reviews, see Chun and Johnson, 2011; Gazzaley and Nobre, 2012). Behaviorally, participants responded faster on Modality retro-cue trials and to a lesser extent on Semantic retro-cue trials than Uninformative cue trials, with no significant RT differences between Semantic and Modality cue trials. This finding provides further evidence that various representational features can be used to selectively access mnemonic representations, in accordance with previous studies (Pertzov et al., 2013; Li and Saiki, 2015; Backer et al., 2015; Ye et al., 2016; Heuer and Schubö, 2016; Niklaus et al., 2017). We observed, however, only a weak effect of retro-cuing on d′ and no effect on accuracy in the present study, likely because of ceiling effects.

### Common neural correlates of reflective attention

A primary goal of this study was to characterize the neural correlates of reflective attention common to both Semantic and Modality (i.e., Informative) retro-cues relative to Uninformative retro-cues. Surprisingly, the univariate fMRI results did not reveal any brain regions that were more active for both Semantic and Modality retro-cues, compared with Uninformative cues. Regarding the MVPA results, pattern activity that reliably discriminated Semantic retro-cues from the other retro-cue conditions did not spatially overlap with pattern activity encoding Modality retro-cues, within the PFC (except for a very small cluster in the left posterior MFG/precentral sulcus). However, during the early phase of the retention interval, regions within the occipital lobe and PPC (including the left and right AG, IPS, and precuneus/SPL) had activation patterns that conveyed information about both the Semantic and Modality retro-cues. These results indicate that PPC regions are involved in top-down orienting of attention to memory, regardless of the feature used to selectively access the retro-cued representations. Ciaramelli et al. (2008) proposed that the posterior IPS is a hub for top-down attentional orienting to episodic memory during retrieval. Notably, the cluster we observed in the left IPS is very close to that suggested by Ciaramelli et al. (2008), as well as to a left IPS cluster observed during perceptual attentional orienting based on spatial and semantic pre-stimulus cues in Cristescu et al. (2006). Taken together, these results support Ciaramelli et al.’s (2008) idea that nearby portions of the posterior IPS may be involved in mediating different forms of attentional control. We discuss the role of AG in reflective attention in a separate section below.

### Neural correlates of semantic-based reflective attention

Overall, Semantic retro-cues were associated with a left-lateralized fronto-parietal network, which included left DMPFC, VLPFC, and DLPFC, and parietal regions spanning the AG, IPS, and SPL/precuneus. Both the univariate analysis and MVPA revealed an area of the left DMPFC that was uniquely associated with Semantic retro-cues, but not Modality or Uninformative retro-cues. These results suggest that this portion of the DMPFC may be involved in retrieving and holding in mind semantic memory. Although all retro-cue conditions rely on semantic knowledge to some extent to interpret the retro-cue, only the Semantic retro-cue requires deeper semantic processing to select the retro-cued representations belonging to the retro-cued category. These findings support Binder et al.’s (2009) proposal that the DMPFC mediates top-down semantic retrieval.

The univariate results further showed that compared with Modality and Uninformative retro-cues, Semantic retro-cues led to greater activation in the left VLPFC. Qualitative inspection of the VLPFC univariate time courses revealed that Semantic retro-cues had the greatest activation, followed by Modality, and finally Uninformative cues. The MVPA results corresponding to the early phase of the retention interval, revealed a cluster within the left VLPFC, whose activation patterns distinguished the Uninformative retro-cue condition from the Informative cue conditions. This VLPFC cluster partially overlapped with the univariate VLPFC cluster.

Considering both the univariate and MVPA results, the left VLPFC mediates a process that is active when accessing a STM representation based on its semantic information, and to a lesser extent, its input modality. In the present task, a Semantic retro-cue requires maintenance of four representations, while accessing semantic memory to categorize each representation as animal or music and updating STM accordingly. Orchestrating these processes requires substantial cognitive control. A similar process is required for a Modality retro-cue except that the sensory modality is intrinsic to the representations and does not have to be internally generated, thereby requiring less cognitive control. The Uninformative retro-cue only requires maintenance of the four representations and no categorization or inhibition of STM representations. Thus, one interpretation is that the left VLPFC mediates a general cognitive control process that enables the top-down selection of task-relevant memory representations and inhibition of task-irrelevant representations (Thompson-Schill et al., 1997; Johnson et al., 2005; Nee and Jonides, 2009; Gazzaley and Nobre, 2012) and is most strongly recruited during semantic-based reflective attention. A second, but not mutually exclusive, interpretation is that Semantic retro-cues lead to stronger engagement of semantic memory and thus greater left anterior VLPFC activation than Modality retro-cues, in line with prior studies demonstrating the VLPFC’s role in semantic memory (Fiez, 1997; Poldrack et al., 1999; Dobbins et al., 2002; Devlin et al., 2003; Badre and Wagner, 2007; Raposo et al., 2009; Han et al., 2012). A third possibility is that the VLPFC is involved in not only cognitive control during semantic processing, but also in the maintenance of semantic information. This interpretation is in accordance with a prior fMRI study demonstrating that a retro-cued visual representation’s category could be decoded from the lateral PFC, but only when the task involved making a categorical judgment (Lee et al., 2013). Future studies are needed to clarify the precise role of the left anterior VLPFC in semantic-based reflective attention.

The univariate results also revealed a relatively large region of the left DLPFC, including a portion of MFG, which was more strongly activated following Semantic than Modality retro-cues. The MVPA results revealed an overlapping cluster within the left DLPFC whose activation patterns discriminated only Modality retro-cues from the other cue conditions. Taken together, these results suggest that the left DLPFC mediates a process that is more similar for Semantic and Uninformative retro-cues than for Semantic and Modality retro-cues. Since the analysis contrasting Intermodal and Intramodal Semantic retro-cue trials did not reveal differences in PFC, the Intermodal Semantic trials (i.e., dividing reflective attention cross-modally) did not drive this effect. An alternative interpretation is that because of the higher cognitive control demands of Semantic retro-cues, they may not have been as effective as the Modality retro-cues in reducing STM load. Consequently, this decreased DLPFC activation following Modality retro-cues may in part reflect reduced load compared with Semantic retro-cues.

The univariate analyses also revealed stronger deactivation on Semantic than Modality retro-cue trials in a variety of regions, including a large bilateral extent of the occipital lobe, right rostro-medial SFG, right mid-cingulate gyrus, and a right lateral cluster encompassing the supramarginal, postcentral, and precentral gyri. Some of these regions overlap with those associated with the default mode network (Shulman et al., 1997; Binder et al., 1999; Raichle et al., 2001). Previous studies have shown that experimental conditions with greater task difficulty are often associated with stronger deactivation in the default mode network (McKiernan et al., 2003; Pallesen et al., 2009; Lin et al., 2011). In the present study, the observed deactivation in widespread areas may reflect a stronger diversion of attentional resources away from perceptual or other irrelevant cortical regions to brain regions involved in higher level attentional selection or semantic memory processing, on semantic retro-cue trials (also see McKiernan et al., 2003). Thus, the present data suggest that orienting attention to memory representations based on their semantic category (which must be abstracted from the stimuli) may be more effortful or demand greater attentional resources than orienting based on a stimulus feature that is inherent in the representations (i.e., input modality).

### Time course of reflective attention

The MVPA results discussed so far were observed during the early phase of the retention interval. Notably, the MVPA corresponding to the late phase did not successfully decode activation patterns related to the Semantic cue condition, unlike the Modality and Uninformative conditions. By a few seconds into the retention interval, participants may have already categorized the STM representations as “animal” or “music,” selected the task-relevant representations, discarded the category information, and were simply maintaining the representations without their semantic category labels until the probe stimulus was presented. Consequently, activation patterns no longer carried information specific to the retro-cued semantic category during the late phase of the retention interval. Furthermore, during the late phase, information related to only the Modality retro-cues was conveyed by activation patterns in the bilateral auditory and visual cortices, which may have reflected maintenance of the retro-cued stimuli and/or anticipation of the probe’s modality (also see Lepsien et al., 2011; Kuo et al., 2014).

### Intramodal versus Intermodal reflective attention

Both the Uninformative and Semantic retro-cues led to greater activity in DLPFC and parietal regions than Modality retro-cues, according to the univariate results. Because participants were instructed to maintain only the retro-cued representations on Modality retro-cue trials, this effect may reflect greater STM load on the Uninformative cue trials (i.e., rehearsing four vs two representations). This interpretation is in accordance with prior research demonstrating that working memory load/capacity modulates activity in the DLPFC and IPS/PPC (Linden et al., 2003; Todd and Marois, 2004; Xu and Chun, 2006; Lepsien et al., 2011; Leung and Alain, 2011; Trapp and Lepsien, 2012). Furthermore, although Semantic retro-cues led to stronger activation in the left VLPFC, IFG, and AG than Uninformative retro-cues, no regions were significantly more activated following Uninformative than Semantic retro-cues, suggesting that Semantic retro-cues may not be as effective as Modality retro-cues in reducing memory load. This pattern of results could also be explained by the fact that both Uninformative and Intermodal Semantic retro-cue trials required participants to divide their attention across auditory and visual representations.

Therefore, we contrasted the Intermodal and Intramodal Semantic retro-cue trials to determine the extent to which cross-modally dividing reflective attention underlies this increased fronto-parietal activation. Notably, this univariate analysis revealed no difference in activation between Intermodal and Intramodal Semantic retro-cue trials in the PFC. Thus, the Intermodal semantic trials did not drive the increased activity observed in the left DLPFC, DMPFC, and VLPFC for Semantic versus Modality retro-cues. Furthermore, the lack of PFC activation differences during cross-modally divided reflective attention differs from several previous studies, using pre-stimulus cues or task instructions to guide participants’ attention during external stimulation, which found increased activity in PFC regions during cross-modally divided attention (Loose et al., 2003; Johnson and Zatorre, 2006; Vohn et al., 2007; Moisala et al., 2015; Salo et al., 2017). Further studies are necessary to determine whether PFC activation, or lack thereof, reflects a fundamental difference between dividing attention across modalities when stimuli are externally present versus when attention is guided to mnemonic representations.

However, the Intermodal trials showed greater bilateral parietal activation, along with longer RTs to the probe stimulus, than the Intramodal trials. We closely examined this Intermodal > Intramodal bilateral parietal activation in relation to the parietal activation observed for Semantic > Modality retro-cues. This revealed overlapping activation in the left IPS and precuneus/SPL, suggesting that these regions are not involved in pure semantic-based reflective attention. Furthermore, a similar examination of the results further revealed that this Intermodal > Intramodal parietal activation also overlapped with that of the Uninformative > Modality contrast, specifically within portions of left and right IPS and precuneus/SPL.

Taken together, these observations suggest that dorsomedial portions of the bilateral PPC are involved in coordinating reflective attentional resources across modalities. One possible mechanism is that this parietal activation indexes cross-modally dividing reflective attention on both Intermodal and Uninformative cue trials, which requires greater attentional demand than when attention is undivided across modalities. This explanation assumes that memory load is reduced to two representations on both Intermodal and Intramodal trials, such that these differences are not because of memory load per se, but rather to differences in managing attentional resources (see also Magen et al., 2009). However, it is also possible that because of the difficulty of coordinating attentional resources across modalities’ STM stores, memory load was not reduced as much on Intermodal trials compared with Intramodal trials. This could explain the behavioral finding that RTs were slower on Intermodal than Intramodal trials, and memory load could still be a contributing factor driving the parietal activation. Alternatively, prior studies have demonstrated that parietal (and prefrontal) cortex may mediate modality-specific STM processing (Kong et al., 2014; Michalka et al., 2015). Consequently, if attention is divided across modalities, then attention to two different STM stores may result in more widespread parietal cortex activation than attention to a single modality, as observed in the present study. Future studies are needed to dissociate these mechanisms.

### The role of the AG in reflective attention

Many of the data analyses revealed significant activation in the inferior parietal lobule, especially within the AG. Notably, the MVPA revealed that activation patterns in bilateral AG discriminated Semantic, as well as Modality retro-cues, from the other cue conditions. These spatially overlapping activation patterns were mostly within the ventral AG and intersected with the left ventrolateral AG ROI identified in Seghier et al. (2010). These findings suggest that the left ventral AG plays a role in reflective attention, regardless of the information used to access a particular memory representation. This specific process may reflect general conceptual identification of STM representations in the present study, for example, determining abstract category (e.g., animal vs music; visual vs auditory) in accordance with Seghier et al. (2010). Alternatively, the ventrolateral AG may be involved in visual imagery, since it was activated more strongly when attending visual STM representations than auditory representations.

We observed a different pattern of results in the left dorsomedial AG. The whole-brain univariate results revealed greater activation for Semantic than Modality retro-cues within this region. However, the Intermodal Semantic trials drove this effect. The whole-brain univariate analysis showed significantly stronger activation for the Intermodal than Intramodal trials in parietal cortex, overlapping with the dorsomedial AG ROI identified in Seghier et al. (2010). Thus, these results support Noonan et al.’s (2013) prediction that increased executive function demands during semantic processing, such as that occurring on Intermodal semantic trials, should lead to greater activation in the dorsal AG.

Notably, the whole-brain univariate results revealed a region in the left dorsal AG, adjacent to the dorsomedial AG ROI and extending laterally, that was uniquely activated for Semantic retro-cues (especially for the univariate Semantic > Modality contrast). This region did not show significant voxels for the Intermodal versus Intramodal contrast. Thus, this dorsolateral region of the left AG must mediate a process specific to semantic-based reflective attention that is insensitive to executive function demands. Unlike sensory modality information that is intrinsic to the memory representation, semantic-based reflective attention requires abstracting higher-level information from the memory representation to categorize it. Thus, it is plausible that this subregion of the left AG is involved in this categorization process and/or works with PFC regions to access semantic memory.

### Conclusion

In conclusion, attention and memory interact continuously during goal-directed behavior. Here, we have provided further evidence supporting the notion that different representational features can be used to selectively access memory representations. The fMRI analyses revealed that regions within the PPC, including portions of the IPS and ventral AG, are involved when selectively accessing representations based on either their input sensory modality or semantic category. These regions may mediate attentional control and/or conceptual processing during reflective attention. The fMRI results also demonstrated that semantic-based reflective attention engages a left-lateralized fronto-parietal network, including the left DLPFC, anterior VLPFC, and DMPFC, as well as dorsal AG, to mediate access to semantic memory and semantic categorization of each memory representation. Finally, the bilateral dorsomedial PPC, specifically portions of the Precuneus/SPL and IPS, are engaged when dividing attention cross-modally within memory. Thus, fronto-parietal regions are flexibly recruited depending on how memory representations are selectively accessed, with additional regions recruited when high-level information (e.g., semantic category) needs to be abstracted from the representations before their attentional selection.

## Synthesis

Reviewing Editor: Bradley Postle, University of Wisconsin Decisions are customarily a result of the Reviewing Editor and the peer reviewers coming together and discussing their recommendations until a consensus is reached. When revisions are invited, a fact-based synthesis statement explaining their decision and outlining what is needed to prepare a revision will be listed below. The following reviewer(s) agreed to reveal their identity: Anastasia Kiyonaga, Nicholas Myers.

Here is the full text of the two reviews:

Reviewer #1

This study examines the fMRI activations associated with retro-cuing short-term memory based on sensory Modality vs. semantic cues. The behavioral and neural consequences of this sort of attentional orienting within working memory have been enthusiastically studied recently, so the general concept is a timely one. I like how the design crosses sensory and semantic cueing methods, and I commend how transparently the methods are described. However, I’m unsure what we can conclude from the current data and analyses. In short, the theoretical background and hypotheses are unclear to me, making it difficult to interpret the sometimes unexpected (or null) findings. I’m not sure the analysis approach is well-suited to tell us something new about these processes, and I think the study could make a more meaningful contribution with additional data/analyses to resolve some current ambiguities.

In the intro, I’m missing a clear description of exactly what problem the study is addressing. It’s also unclear to me what aspects of this study are novel vs. replicating or combining findings/manipulation that already exist. E.g., the intro says prior studies have demonstrated that the neural substrates of reflective attention vary as a function of the object of attention, as well as that spatial vs. semantic retro-cuing during an auditory task shows domain-specific EEG modulations. However, the intro doesn’t specify what the differences were in either case, and left me wondering exactly what motivated the current study. It’s unclear to me if specific theories or hypotheses are being tested/compared, or if this is just an exploration of whether the BOLD responses to these conditions will be the same or different. The intro makes a broad prediction that sensory vs. semantic cuing may be different, but doesn’t specify in what way, or what it will tell us if they are or aren’t.

Much of the above could be resolved with revisions to the writing, but the issue here is that this lack of a strong theoretical framework, or a clear set of hypotheses, makes me unable to sort all the results in a meaningful way. Moreover, when expected effects were absent (e.g., Modality > Uninformative; Uninformative > semantic), it’s tough to say whether that is an interesting novel outcome, or a consequence of too few participants/too little data. I have no major issues with the design. I just can’t tell exactly what we learn from it, and I think the study needs to go further to examine what the observed activations mean. For instance, the discussion refers to the idea that multivariate analyses may be more sensitive to detect meaningful differences that are absent in some univariate contrasts-why not do those analyses? A searchlight analysis could examine which regions discriminate between the cueing domains, or and ROI-based approach could examine if evidence for auditory vs. visual, or animal vs. musical differed in relevant regions like the lateral PFC. I don’t mean to push a more complicated analysis approach if the question can be well-addressed with something more straightforward. But in this case, the expected findings seem to simply replicate previous findings, and the unexpected findings are somewhat perplexing. Thus some competing interpretations could be addressed with additional analyses. Some effort could also be made to relate the fMRI activations to behavior, which may further clarify the role that those activations play.

Minor:

-The abstract alludes to an interaction between representational features and attentional demand, but I’m not sure where that evidence comes from

-the use of the term “domain” can be confusing here, cause it can be unclear whether that refers to the sensory Modality, or some other feature

-p. 14, lines 294-296-the justification for this negative volume thing was confusing to me

-you might consider also posting unthresholded maps on a repository (like neurovault), rather than just all the tables

-where is the conjunction analysis that the discussion refers to? is this even a valid analysis when there is no Modality > Uninformative effect to speak of?

Reviewer #2

Summary

This study used fMRI to investigate selection of items in working memory. In particular, the study compared the effects of two different kinds of retrospective cues: Modality cues indicated whether the visually or the aurally presented items would be probed, while semantic cues indicated whether items from one or another semantic category would be probed. While the majority of analyses and conclusions seem sound, I have a few unresolved questions about the lack of a cueing effect in the semantic cue condition and the interpretation of some of the results.

Major Points

1. Could semantic vs. Modality cue trials have differed in probe expectation? I.e., Modality cues also indicated which Modality the probe stimulus would be from, so neural effects could be related to anticipation of that specific Modality. Would this change the interpretation of any results?

2. The behavioural effects of the semantic cues were very small - is it possible that participants did not use these to the same extent as the Modality cues? Could this have influenced the neural results? This would also mean that the effective load is not reduced (as much) on semantic cue trials.

3. I don’t follow the argument that there were domain-general effects of cueing in this study. Would this not require a conjunction analysis showing voxels in which both Modality cues and semantic cues lead to higher activation than Uninformative cues?

Minor Points

4. The semantic vs Modality cue contrast differs both in the cue type and the cued content. However, these two could be dissociated by contrasting Modality cues with the within-Modality semantic cues - would this not permit looking at cue effects while keeping the cued content constant?

5. Several sections in the discussion are fairly speculative (e.g., p. 23: ‘it is possible that this process of inhibiting ...’, p. 29: ‘Perhaps this reflects a fundamental difference ...’) , or based on reverse inference (p. 24: anterior vlpfc ‘plays a role in semantic/conceptual processing’), so their contribution to the overall conclusions seems a bit limited. Perhaps some of these sections could be shortened or removed?

6. I did not quite understand this claim in the discussion (p. 27): ‘we did not observe distinct brain regions activated following only Semantic or Modality retro-cues.’ - Is this not what the Semantic vs. Modality cue contrast showed?

7. The discussion draws links between internal attention (this study) and external attention, which was not studied here. This comparison seems tricky, given that there were no external attention cues in the current study.

8. Is it standard practice to smooth functional images before coregistering them to thee anatomical image? Wouldn’t this decrease the accuracy of the coregistration?

9. From what I can tell, items always appeared in the same array, raising the possibility of learning across repeated exposures, effectively reducing the memory load via chunking. Is there a way to test for this?

10. Is it worth relating the present results to the study by Lee et al., which found semantic-level representation of WM contents in ventrolateral PFC? Lee, S. H., Kravitz, D. J., & Baker, C. I. (2013). Goal-dependent dissociation of visual and prefrontal cortices during working memory. Nature neuroscience, 16(8), 997.

Bo-Cheng Kuo has also done a number of studies showing the relevance of inferior frontal cortex to retrocueing, e.g.:

Kuo, B. C., Stokes, M. G., Murray, A. M., & Nobre, A. C. (2014). Attention biases visual activity in visual short-term memory. Journal of cognitive neuroscience, 26(7), 1377-1389.

I wonder if this study is worth citing?
